# Mitotic cells contract actomyosin cortex and generate pressure to round against or escape epithelial confinement

**DOI:** 10.1038/ncomms9872

**Published:** 2015-11-25

**Authors:** Barbara Sorce, Carlos Escobedo, Yusuke Toyoda, Martin P. Stewart, Cedric J. Cattin, Richard Newton, Indranil Banerjee, Alexander Stettler, Botond Roska, Suzanne Eaton, Anthony A. Hyman, Andreas Hierlemann, Daniel J. Müller

**Affiliations:** 1 Department of Biosystems Science and Engineering, Eidgenössische Technische Hochschule (ETH) Zurich, Mattenstrasse 26, Basel 4058, Switzerland; 2 Department of Chemical Engineering, Queen's University, 19 Division Street, Kingston, Ontario, Canada K7L 3N6; 3 Max Planck Institute of Molecular Cell Biology and Genetics, Pfotenhauerstrasse 108, 01307 Dresden, Germany; 4 Department of Chemical Engineering, Massachusetts Institute of Technology (MIT), 500 Main Street, Cambridge, Massachusetts 02139-4307, USA; 5 The David H. Koch Institute for Integrative Cancer Research, 500 Main Street, Cambridge, Massachusetts 02139-4307, USA; 6 Neural Circuit Laboratories, Friedrich Miescher Institute (FMI) for Biomedical Research, Maulbeerstrasse 66, Basel 4058, Switzerland

## Abstract

Little is known about how mitotic cells round against epithelial confinement. Here, we engineer micropillar arrays that subject cells to lateral mechanical confinement similar to that experienced in epithelia. If generating sufficient force to deform the pillars, rounding epithelial (MDCK) cells can create space to divide. However, if mitotic cells cannot create sufficient space, their rounding force, which is generated by actomyosin contraction and hydrostatic pressure, pushes the cell out of confinement. After conducting mitosis in an unperturbed manner, both daughter cells return to the confinement of the pillars. Cells that cannot round against nor escape confinement cannot orient their mitotic spindles and more likely undergo apoptosis. The results highlight how spatially constrained epithelial cells prepare for mitosis: either they are strong enough to round up or they must escape. The ability to escape from confinement and reintegrate after mitosis appears to be a basic property of epithelial cells.

## Abstract (web summary)

In epithelial layers cells must round up prior to division. Here the authors use micropillar arrays to mimic epithelial confinement and show that MDCK cells generate force to create space to divide; if unable to generate sufficient force they escape the micropillars to divide and return to confinement.

## Graphical abstract

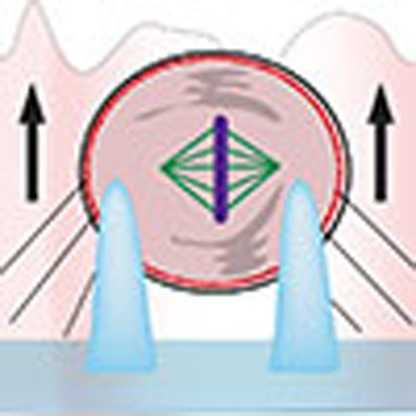

At the beginning of mitosis, cells markedly change their morphology as they round up^1^,^2^. During mitotic cell rounding, the microtubule cytoskeleton forms the mitotic spindle, a central machinery that captures and organizes chromosomes^3^,^4^. Mitotic cell rounding occurs in the vast majority of animal cells^1^,^5^ and plays a role in maintaining tissue organization^2^,^6^,^7^,^8^,^9^,^10^. It is now clear from studies in tissue culture that cell rounding is driven by the contraction of the actomyosin cortex and associated proteins^4^,^6^,^10^,^11^,^12^,^13^. The cortex can only produce contractile forces and mitotic cells also generate an outward force by the modulation of intracellular pressure, which is governed by plasma membrane transporters^14^. Together, these mechanisms lead to an ∼10-fold increase in cortex tension and hydrostatic pressure as cells progress through mitosis^14^,^15^. Recent studies have revealed that the generation of cell cortex contraction and tension directly correlates with the accumulation of active myosin II at the cortex^16^. The master regulator of mitosis, cyclin-dependent kinase 1, balances cell cortex tension and hydrostatic pressure by using RhoA kinase to stimulate and p21-activated kinases to suppress myosin II recruitment to the cortex.

While previous *in vitro* studies provide valuable insight into the mechanism of cell rounding, they do not fully describe the rounding of cells *in vivo*. Cells *in vivo* are spatially confined in more than one dimension by other cells and surrounding tissue and, to round, a mitotic cell must exert force^9^,^17^,^18^,^19^. The mechanisms of cell rounding in the confinement of tissue are not well studied. Cell culture studies indicate that the loss of substrate adhesion is sufficient for the rounding of isolated cells^20^, but that actomyosin cortex contraction and the accompanying increase in intracellular pressure are required for the generation of rounding forces against confining structures^14^,^21^,^22^.

Cell rounding under confinement is particularly relevant to cell division in an epithelium. Epithelia comprise densely packed layers of cells that are organized into sheets. These sheets form tissues such as the epidermis, the surfaces of the eye and the surfaces of the hollow tubes and sacs that make up the digestive, respiratory, reproductive and urinary tracts. Tightly packed epithelial cells secrete an extracellular matrix called the basal lamina, which anchors the epithelial tissue to the basement membrane. This membrane acts as a scaffold on which epithelial cells can grow and regenerate after injury. Epithelia fulfil a variety of functions including protection, absorption, sensory reception and secretion. Tight junctions between cells enable epithelial layers to act as effective mechanical barriers^23^,^24^. If epithelial layers are damaged, their protective role is compromised which may result in problems in tissue development and regeneration or the occurrence of diseases such as cancer^25^,^26^,^27^. It has been shown that epithelial cells rounding for mitosis regulate adhesion and orient their spindle axes^28^,^29^. Epithelial cells that cannot round for mitosis cannot properly orient and assemble their mitotic spindle, which can lead to their mislocalization within the tissue and eventually to apoptosis, cancer or other disease states^7^,^18^,^30^. Despite our understanding of the role and importance of epithelia, the mechanisms governing the rounding of epithelial cells for mitosis and their influence on cell division have not yet been fully described.

Cells *in vivo* continually encounter and respond to a multitude of environmental stimuli. While the role of biochemical signals has long been appreciated, the importance of mechanical signals has only recently begun to be investigated^31^,^32^,^33^. The extracellular matrix and adjacent cells can impart such mechanical cues. Microfabrication technologies have enabled the production of microscale topographies to study the effect of mechanical cues on cellular function at the cell–substrate interface^34^,^35^,^36^,^37^. Devices featuring channels, structured substrates, slits, cantilevers and pillars can be fabricated to such an end. Of particular interest are arrays of micropillars that can be used to investigate forces generated by cell adhesion, migration and differentiation at subcellular scales^38^,^39^,^40^,^41^. Analysing the deflection of micropillars of known geometry and dimensions in response to cell-generated forces allows the quantification of these forces and sheds light on the dynamic processes of adhesion, differentiation and mechanotransduction. To measure mechanical forces at the subcellular level in these applications, the micropillar spacing must be much smaller compared with cellular dimensions. Until now, however, micropillar arrays that mimic the mechanical constraints of the epithelia have not been introduced.

Here we introduce micropillar arrays with spatial and mechanical properties designed to impose lateral confinement on epithelial cells similar to that which they would experience normally in monolayers. For our studies we use Madin–Darby canine kidney (MDCK) cell lines that are commonly used as a model for epithelial cells^11^,^42^. We observe that MDCK cells deflect nearby micropillars as they round and thereby create sufficient space for mitosis^21^,^22^. Optically analysing the deflection of the individual confining micropillars enables us to quantify the rounding force generated by individual mitotic cells. By specific chemical perturbation, we confirm that this rounding force is generated by an inward-directed actomyosin contraction and an outward-directed hydrostatic (for example, osmotic) pressure^14^,^21^,^22^. If micropillar separation does not allow sufficient space for mitotic cell rounding, cells travel up the pillars to escape confinement, driven by actomyosin contraction and the hydrostatic pressure generated by the rounding mitotic cell. Once freed from confinement, these cells conduct mitosis in an unperturbed manner. After mitosis, the daughter cells return to spaces between the confining micropillars. Mitotic cells unable to escape spatial confinement cannot orient their microtubule spindle properly and are more likely to undergo apoptosis.

## Results

### Mitotic cells round against adjacent cells and change height

**Figure 1:**
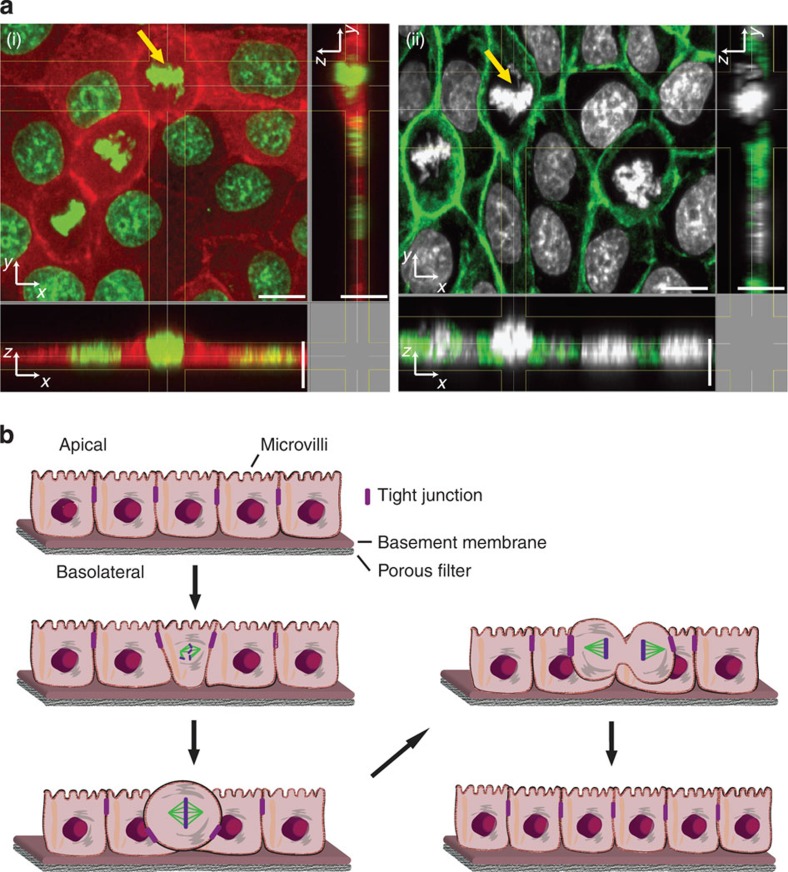
Mitosis in polarized MDCK cells. (**a**) (i) MDCK cells expressing actin-mCherry (red) and eGFP-labelled histones (H2B-eGFP, green) and (ii) MDCK cells expressing E-cadherin-eGFP (green) and nuclei stained with Hoechst 33342 (grey). Cells were plated on permeable filter supports and grown for 3 days before data acquisition (see Supplementary Fig. 1 for MDCK cells grown for 14 days). The eGFP-labelled histones allowed us to determine the mitotic phases of MDCK cells (see Methods). (i) Confocal microscopy images of polarized MDCK cells expressing actin-mCherry and H2B-eGFP showing mitotic cells rounded up in metaphase (yellow arrow). (ii) Confocal microscopy image of MDCK cells expressing E-cadherin-eGFP that show extensive contacts between metaphase (yellow arrow) and interphase cells. (**b**) Schematic drawing of cell division in the epithelial layer. Scale bars, 10 μm.

We wanted to characterize morphological changes of mitotic cells in the epithelium. As an established epithelial *in vitro* model, we chose MDCK cells. When cultivated on membrane supports, MDCK cells differentiate in a cuboidal epithelial layer^28^ and retain properties characteristic of kidney epithelial cells, such as tight junctions and distinct basal and apical membrane domains^20^,^29^. Using confocal microscopy we monitored mitotic MDCK cells in the epithelial layer (Fig. 1). We followed the mitotic state by expressing enhanced green fluorescent protein (eGFP)-labelled histones (H2B-eGFP), the actomyosin cortex by expressing actin-mCherry and cell junctions by expressing eGFP-labelled E-cadherin. Confocal microscopy images show epithelial mitotic cells rounding and increasing in height by ≈5 μm while retaining tight junctions. Mitotic rounding was observed for MDCK cells plated on permeable filter supports and grown for 3 days (Fig. 1a) and 14 days (Supplementary Fig. 1). This observation is in accord with previous reports that mitotic epithelial cells lose their cuboidal architecture and round towards the apical surface of the epithelia^20^,^28^. Furthermore, the confocal images reveal that mitotic cells mechanically deform adjacent cells and orient their metaphase plate perpendicular to the substrate while rounding. Having observed that mitotic MDCK cells round in the epithelial layer, increase in height and deform adjacent cells (Fig. 1b), we sought to answer the following questions regarding these processes: what is the driving force behind mitotic MDCK cells rounding against the confinement of the epithelium? By which mechanisms do mitotic MDCK cells increase in height in the epithelium? What role does mechanical confinement play in epithelial cells conducting mitosis?

### Engineering micropillars to mimic epithelial confinement

Having found that mitotic cells within the epithelium round against mechanical confinement, we searched for a suitable approach to characterize the interplay between mitotic cell rounding and mechanical confinement. We decided to engineer micropillar arrays to mimic epithelial confinement using soft-lithography methods (Supplementary Fig. 2). The arrays comprised dense hexagonally arranged and flexible poly(dimethylsiloxane) (PDMS) micropillars, which differed in spacing and length (Supplementary Fig. 2b). Interpillar distances of ≈6.8 μm, ≈8.8 μm and ≈14.3 μm were chosen to fit within the range of lateral distances observed between MDCK cells in epithelia (Fig. 1). On average, the slightly conical micropillars had diameters of ≈2 μm at their tip and ≈5 μm at their base. The micropillars lengths ranged from ≈8.2 to ≈13.7 μm as to be comparable to the thickness of the MDCK epithelium (Fig. 1). Different interpillar distances were used to mimic different spatial confinements of epithelial cells and different micropillar lengths were used to mimic different substrate morphologies and rigidities. MDCK cells cultured for up to 14 days on these micropillar arrays polarized and formed extensive junctional contacts similar as to when cultivated on membrane supports (Supplementary Fig. 3). In the following section, we apply the micropillar arrays to characterize the forces generated by single-rounding mitotic cells.

### Measuring forces generated by mitotic MDCK and HeLa cells

**Figure 2:**
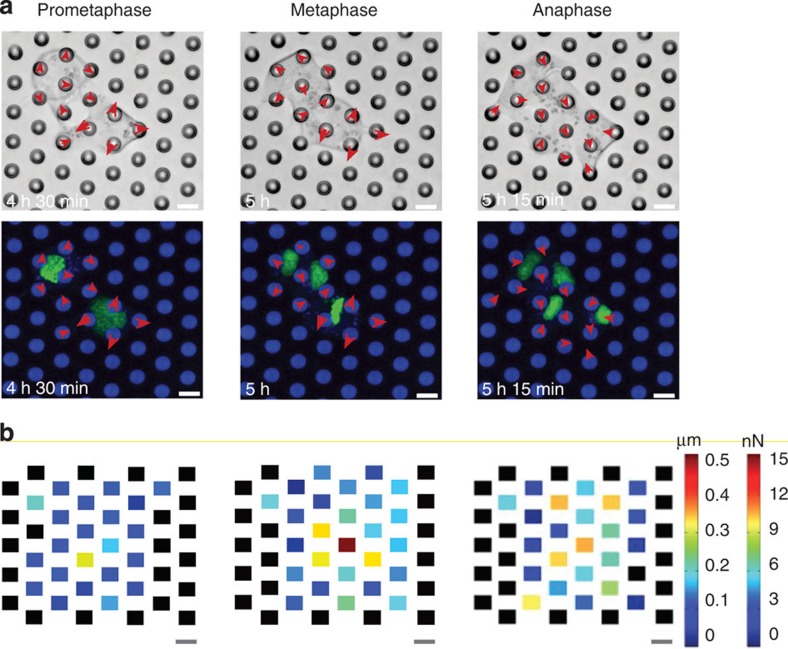
Micropillar deflection reveals the force generated by rounding mitotic MDCK cells. (**a**) Differential interference contrast (DIC) (top) and confocal (bottom) microscopy of MDCK cells expressing H2B-eGFP grown between micropillars having an average distance of ≈14.3 μm and length of ≈13.7 μm (Supplementary Fig. 2). When entering mitosis, the rounding MDCK cells deflect adjacent micropillars (red arrows) until cell division has been conducted. Confocal images show H2B-eGFP in green and DiD-labelled micropillars in blue. Shown are MDCK cells progressing from prometaphase to metaphase and anaphase. (**b**) Micropillar deflection and force applied by mitotic cells shown in **a**. For determination of the micropillar spring constant, see Supplementary Fig. 4 and for micropillar deflection and force, see Supplementary Fig. 5. Scale bars, 10 μm.

Different micropillar spacing and lengths determine the degree to which pillars bend in response to the force generated by rounding mitotic cells. To be able to quantify these forces, we determined the spring constant of the micropillars using finite-element analysis and by atomic force microscopy (AFM) (Supplementary Fig. 4). The spring constants ranged from 42.9 to 29.0 mN m^–1^ for 8.2- and 13.7-μm-long micropillars, respectively. After determining the spring constants, we seeded MDCK cells onto the micropillar arrays with an interpillar distance of 14.3 μm. Using confocal microscopy, we followed a single histone eGFP-labelled (H2B-eGFP) MDCK cell progressing through mitosis and recorded the deflection of individual micropillars (Fig. 2a; Supplementary Fig. 5). To convert these deflections into forces, we assumed that the rounding cells most efficiently deflected the micropillars when pushing against their upper end (Supplementary Fig. 4). We multiplied the micropillar deflections with the micropillar spring constants to quantify the rounding forces the mitotic cells generated in prometaphase, metaphase and anaphase (Fig. 2b). These force maps quantified the forces generated by single rounding mitotic MDCK cells. When looking at the individual pillars surrounding a mitotic cell, it becomes evident that not every pillar experienced the same force, with forces ranging from 3 to 15 nN (Fig. 2b). We attribute this asymmetric force distribution to the fact that the mitotic cell was not always placed exactly between the surrounding micropillars. Next, we questioned whether the observed deflection of single micropillars occurs uniquely for MDCK cells or is more general. Therefore, we repeated the described experiment using HeLa cells (Supplementary Fig. 6). Similarly to MDCK cells, mitotic HeLa cells deflected the micropillars and generated forces of up to 15 nN.

We have previously shown that in mitotic HeLa cells, the actomyosin cortex contracts and hydrostatic pressure increases to generate an outward-directed rounding force against extracellular confinements^21^. We used the AFM-based constant height (10 μm) assay previously applied to characterize mitotic HeLa cells to measure the rounding force and pressure of uniaxially confined MDCK cells (Supplementary Fig. 7)^14^,^15^. Mitotic MDCK cells generated an average force of ≈43 nN and pressure of ≈310 Pa to round against the mechanical confinement of the AFM cantilever. As the AFM-based assay constrains the mitotic cell differently (that is, uniaxial confinement) and because the contact area between the AFM cantilever and cell is greater than between micropillars and cell, the rounding force measured by AFM was significantly higher. Most importantly, however, AFM measurements qualitatively confirmed our quantitative force measurements made with the micropillar array.

### Screening proteins contributing to mitotic rounding forces

**Figure 3:**
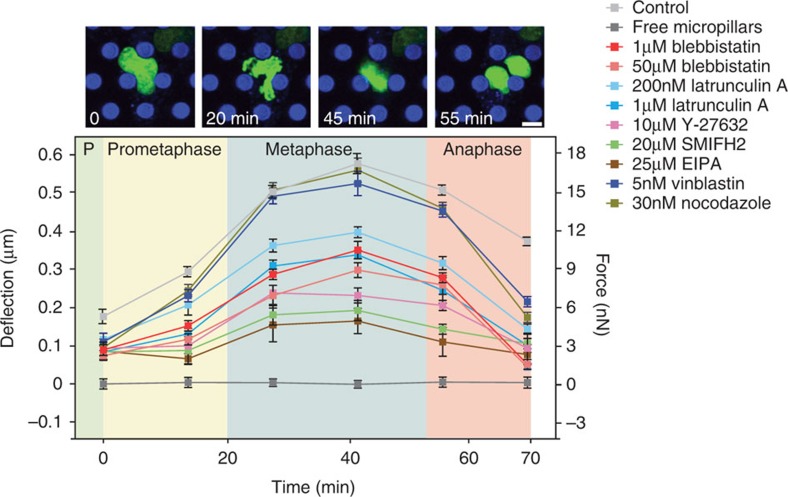
Forces generated by MDCK cells progressing through mitosis that are confined by micropillars. Micropillar deflection and force generated by rounding mitotic MDCK cells increase from prophase to prometaphase until they reach maximum values in metaphase. In anaphase, micropillar deflection and, thus, the rounding force drops significantly. The generation of rounding force by mitotic MDCK cells was perturbed by the chemicals indicated. ‘Control' indicates pillar deflection measured in absence of any chemical perturbation and ‘free micropillars' indicates pillar deflection in absence of cells (that is, experimental noise). Fluorescence images show overlaid signals recorded from eGFP-labelled histones (H2B-eGFP, green) and DiD-labelled micropillars (blue) and were taken at the times indicated. Time zero denotes nuclear envelope breakdown. Background of the graph is colored to indicate the time range in which an example cell progresses through prophase (P, green), prometaphase (yellow), metaphase (blue) and anaphase (pink). Micropillars had an average distance of 14.3 μm and length of 13.7 μm. For each condition, the average and s.e.m. of the micropillar deflection as measured for *n*=30 cells is given. Scale bar, 8 μm. Perturbants are described in Supplementary Table 1.

After having shown that the micropillar arrays can be employed to detect the rounding force generated by mitotic cells, we decided to investigate the origin of the rounding forces. To study the role of the actomyosin cortex in generating the rounding force of a mitotic MDCK cell, we perturbed non-muscle myosin II with blebbistatin, actin polymerization with latrunculin A, rho-associated protein kinase ROCK with Y-27632 and hydrostatic pressure with ethylisopropylamiloride (EIPA) or ouabain (Fig. 3; Supplementary Fig. 8). To further characterize the role of F-actin, we inhibited its nucleation factor formin with SMIFH2. The results show that perturbing the actomyosin cortex or the membrane proteins contributing to the osmotic gradient (that is, hydrostatic pressure) directly affects the rounding force generated by the mitotic MDCK cell. Perturbing the mitotic spindle (microtubule) using vinblastine or nocodazole did not affect the rounding force.

In summary, perturbing either the osmotic gradient or the actomyosin cortex influenced the rounding force generated by the mitotic cell. These results were similar to those observed for mitotic HeLa cells, where it was reported that hydrostatic pressure is balanced by the contractile actomyosin cortex to generate an outward-directed rounding force and pressure^14^,^21^. In fact repeating the micropillar experiments using HeLa cells showed similar results to those observed for MDCK cells (Supplementary Fig. 9; Fig. 3). Thus, using the micropillar assay we could show that MDCK cells, similarly to HeLa cells, create an outward-directed hydrostatic pressure and balance this pressure by contraction of the actomyosin cortex to round for mitosis. Together hydrostatic pressure and actomyosin contraction allow the cell to round against the mechanical confinement of the micropillars.

### Cells escape too narrow confinements to conduct mitosis

**Figure 4:**
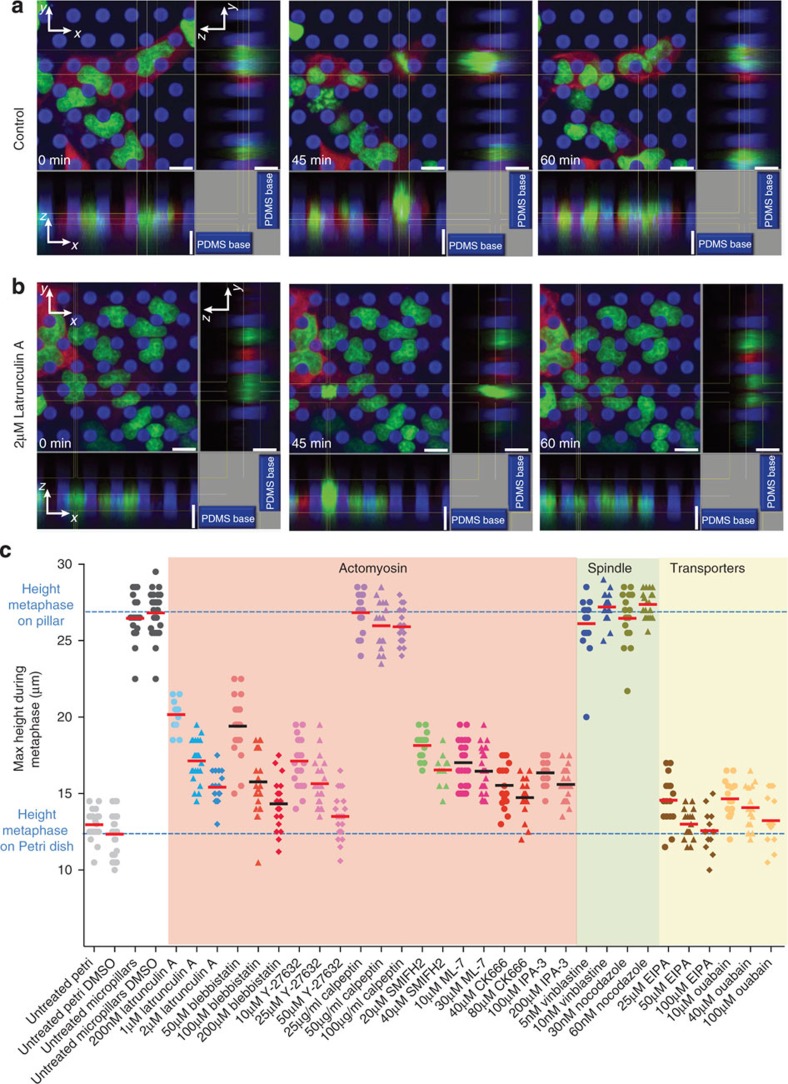
Height elevation of rounding mitotic MDCK cells confined by narrowly spaced micropillars depends on actomyosin cortex and hydrostatic pressure. (**a**) Confocal fluorescence microscopy of MDCK cells expressing actin-mCherry (red) and H2B-eGFP (green) grown on DiD-labelled micropillar arrays (blue). Micropillars had an average distance of ≈6.8 μm and length of ≈8.2 μm. Upon entering mitosis, rounding MDCK cells travel up the micropillars until they protrude above the micropillar apices. After division, daughter cells move back between the micropillars. (**b**) Confocal microscopy of MDCK cells expressing actin-mCherry and H2B-eGFP grown on the micropillar array in the presence of 2 μM latrunculin A. Perturbed mitotic cells cannot travel up the micropillars and remain in space confined by micropillars. Scale bars, 6 μm. (**c**) Perturbations were tested using a micropillar array with ≈6.8 μm interpillar distance. Maximum heights from the substrate to the top of metaphase cells were recorded using confocal fluorescence microscopy (see Methods). The blue dashed lines give the average maximum height of non-perturbed metaphase cells on micropillar substrates and Petri dishes as references. Perturbations are categorized into those affecting the actomyosin (pink background), microtubule spindle (green background) and transporters generating a hydrostatic pressure of the mitotic cell (yellow background). Perturbants were added 20 min before cells entered prophase except for EIPA and ouabain, which were added during prophase because they inhibit the G2/M transition. Each dot or triangle represents one experimentally characterized cell. For each condition, the bar denotes the average. Perturbants are described in Supplementary Table 1.

In the previous section, we described how mitotic epithelial cells generate an outward-directed force to round against mechanical confinement. Next, we questioned what would happen if confinement prevented the cell from creating sufficient space to round for mitosis. As the average diameter of polarized MDCK cells was above 10 μm (Fig. 1), we plated MDCK cells onto micropillar arrays with much smaller interpillar distances of ≈6.8 μm, and monitored the cells progressing through mitosis using confocal microscopy (Fig. 4a). The MDCK cells, initially occupying space between pillars, travelled up the pillars when entering mitosis such that they rounded mostly outside of the confinement imposed by the pillars. After successful mitosis, the two daughter cells moved back between the micropillars. Although mitotic MDCK cells also rounded and travelled up micropillars spaced ≈14.3 μm apart, the phenomenon was much more pronounced on arrays with reduced interpillar spacing of ≈6.8 μm (Supplementary Fig. 10a,b). This indicates that epithelial cells can escape when confinement is too restrictive and after escaping the confinement round-up to conduct mitosis.

To probe the mechanisms driving the mitotic MDCK cell out of mechanical confinement, we repeated the micropillar experiments in the presence of chemical perturbants (Fig. 4b,c). Perturbing the actomyosin cortex with lantrunculin A, blebbistatin, Y-27632, SMIFH2, ML-7, CK666 or IPA3 hinders the ability of mitotic cells to travel up pillars and escape mechanical confinement (Fig. 4b; Supplementary Fig. 10c; Supplementary Table 1). Mitotic cells were similarly hindered when we perturbed their ability to generate hydrostatic pressure with EIPA and ouabain (Fig. 4b; Supplementary Table 1). Together the experiments show that mitotic cells with a compromised actomyosin cortex and/or impaired ability to generate hydrostatic pressure cannot escape mechanical confinement. In support of these observations, further contracting the actomyosin cortex with calpeptin to increase the rounding pressure caused mitotic MDCK cells to move up the micropillars^43^. Perturbation of the mitotic spindle (tubulin) did not affect the ability of mitotic cells to escape confinement.

We further asked whether the upward movement to escape confinement is unique to MDCK cells or can be observed for other cell lines. We repeated the micropillar experiments as described using HeLa cells (Supplementary Figs 11 and 12). Similarly to MDCK cells (Supplementary Fig. 10), mitotic HeLa cells travelled up micropillars if confined by too densely spaced micropillars. However, mitotic HeLa cells could not move up the micropillars if their actomyosin cortex and/or ability to generate hydrostatic pressure were perturbed. These results show that perturbing the proteins involved in generating the rounding force prevents mitotic cells from changing shape against mechanical constraints and precludes their escape from constraints that are too constrictive.

### Cells that cannot escape suffer from reduced viability

**Figure 5:**
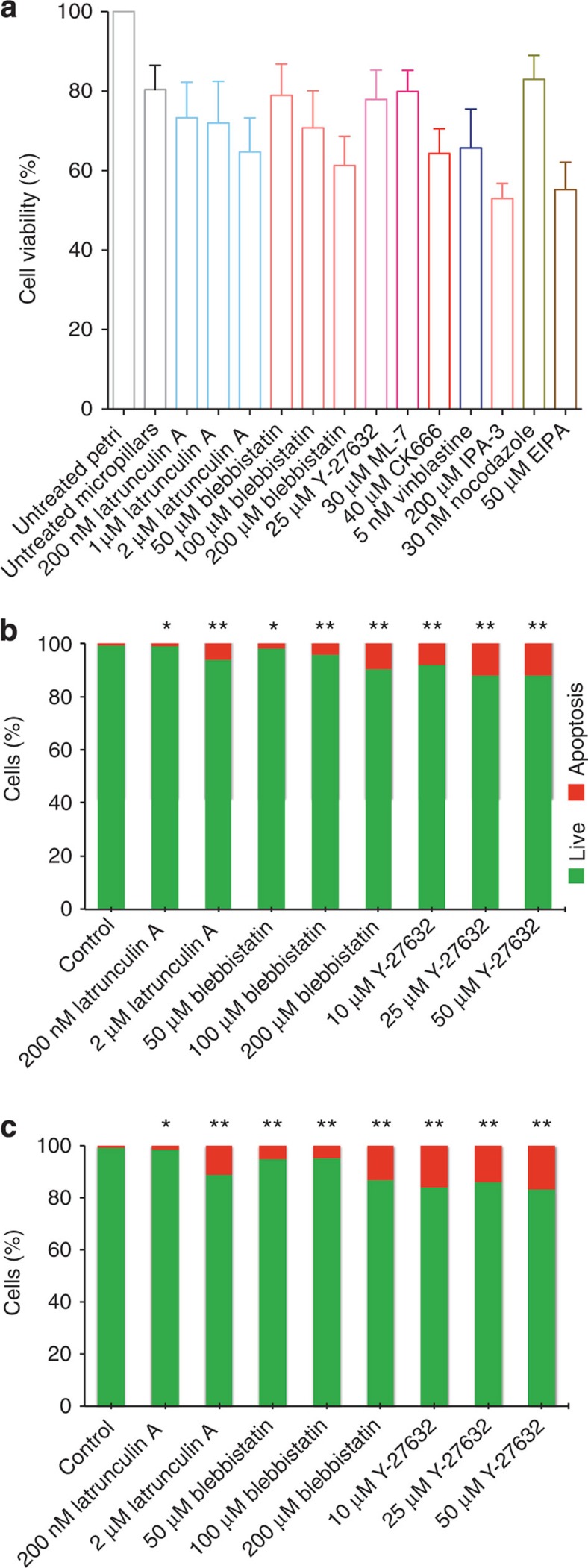
Viability and apoptosis of MDCK cells confined by micropillars. (**a**) 3-(4,5-dimethylthiazol-2-yl)-2,5-diphenyltetrazolium bromide (MTT) assay of MDCK cells cultured on micropillar arrays for 3 h in the presence of different chemical compounds that perturb cellular processes. For each condition, mean and s.d. are given. (**b**) Percentage of apoptotic (red) and living (green) cells on the flat surface in the presence of different perturbants (*n*_cells_=120, 140, 80, 95, 100, 113, 105, 95, 80). (**c**) Percentage of apoptotic and living cells on micropillar arrays (*n*_cells_=200, 150, 180, 210, 220, 150, 170, 210, 170). Apoptotic and living cells were quantified using CellProfiler (see Methods). Micropillar arrays used had average interpillar distances of ≈6.8 μm. Mann–Whitney *P* values indicate the significance relative to the control (untreated) cells. *P<0.01, **P<0.001. Perturbants are described in Supplementary Table 1.

We have observed that mitotic cells generate an outward-directed force to round up against mechanical confinement. If mechanical confinement is too great, mitotic cells employ this rounding force to escape the confinement. We next sought to study the consequences in cases where mitotic cells were unable to escape the confinement of the micropillars. Therefore, we perturbed the actomyosin cortex or the hydrostatic pressure of MDCK cells seeded on cell culture plates or on micropillar arrays with interpillar distance of ≈6.8 μm and characterized cell viability and apoptosis rates (Fig. 5). The direct comparison of MDCK cells being perturbed in the absence and presence of pillars, showed that MDCK cells that cannot escape too narrow micropillar confinements are more likely to go through apoptosis.

### Spindle orientation depends on mechanical confinement

**Figure 6:**
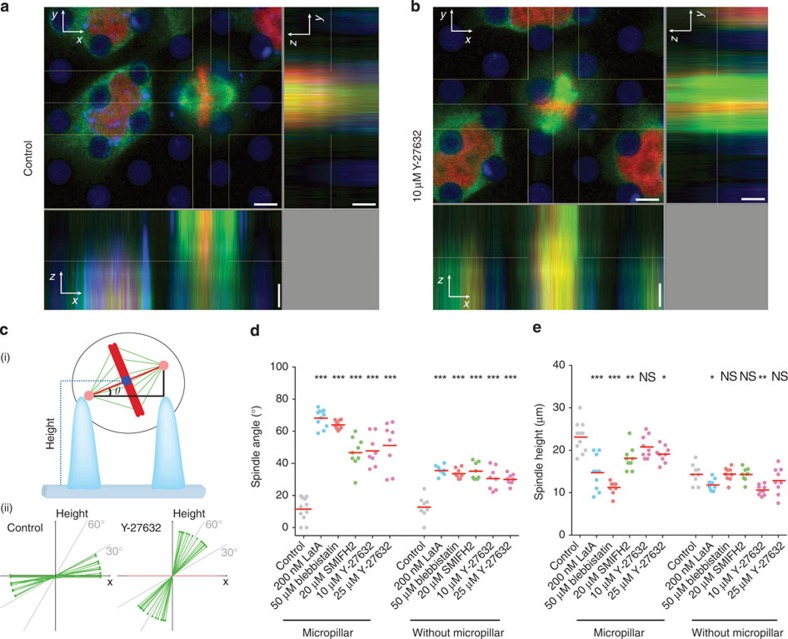
Effect of micropillar confinement on the orientation of the mitotic spindle. To assess the angle and height of the mitotic spindle, H2B-mCherry (red)- and tubulin-eGFP (green)-expressing HeLa cells were cultured on DiD-labelled micropillar arrays (blue) and cell culture plates. Micropillars had average distances of ≈6.8 μm and lengths of ≈8.2 μm. Confocal images were taken of HeLa cells in metaphase in (**a**) absence or (**b**) presence of 10 μM Y-27632. Lateral scale bars, 3.5 μm and vertical scale bars, 5 μm. (**c**) Determination of the spindle angle and height of a confined mitotic cell (i) showed that unperturbed HeLa cells orient their spindle parallel (towards an angle *θ* of 0°) to the substrate plane, (ii) whereas cells cultured in the presence of 25 μM Y-27632 possessed a more vertical orientation (towards an angle *θ* of 90°) of the mitotic spindle (ii). The angle (**d**) and the height (**e**) of the mitotic spindle were influenced by chemical compounds perturbing the actomyosin cortex. The confining micropillars enhanced the perturbation of the mitotic spindle angle and height. Each dot represents one experimentally characterized cell. For each condition, the bar denotes the average. Mann–Whitney *P* values indicate the significance relative to the control (untreated) cells. NS, not significant, *P≥*0.05, *P<0.05, **P<0.01, ***P<0.001. Perturbants are described in Supplementary Table 1.

In the previous section, we observed that mitotic cells that cannot round in an unperturbed manner suffer from reduced viability and are more likely to undergo apoptosis. Next, we questioned the origin of this impaired survival. As a mitotic cell rounds, its microtubule cytoskeleton forms the mitotic spindle^3^,^4^. In epithelia, spindle orientation plays a vital role in ensuring tissue growth and integrity^26^,^30^. Spindle orientation determines the plane of cytokinesis and thus the relative position of daughter cells after mitosis. It has been suggested that spindle alignment parallel to the plane of epithelial tissue promotes tissue growth and spreading, whereas perpendicular spindle alignment results in improperly localized daughter cells, which can in turn lead to defects in tissue morphogenesis^18^,^44^,^45^. Thus, it may be posited that the impaired survival rate observed for mechanically confined mitotic cells is somehow connected to an impaired spindle orientation^46^. To study a possible relationship, we used confocal microscopy and characterized the orientation of the mitotic spindle of cells that could and cells that could not escape the mechanical confinement imposed by micropillars (Fig. 6). In these studies, we used HeLa cells expressing mCherry-labelled histones (H2B-mCherry) and eGFP-labelled tubulin (tubulin-eGFP). Unfortunately, we were unable to express both constructs in MDCK cells. However, since mitotic MDCK and HeLa cells constrained by micropillars showed the same behaviour in all other experiments, we speculate that mitotic spindle alignment in response to mechanical confinement would also be similar in both cell lines. The experiments reveal that HeLa cells, which can escape the mechanical confinement and round up unperturbed orient their mitotic spindle parallel to the substrate. Mitotic cells that cannot deform or escape the micropillar confinement show an impaired spindle orientation (Fig. 6b–e). Thus, when trapped within too great mechanical confinement, mitotic cells could not properly orient the spindle as they do in the non-confined case.

## Discussion

**Figure 7:**
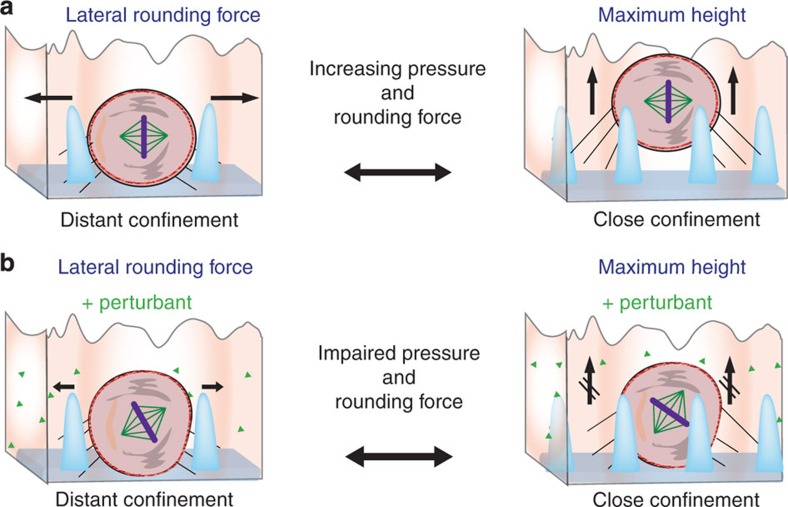
Proposed model of mitotic cell rounding in confinement. (**a**) Mitotic epithelial (MDCK) and other animal (HeLa) cells generate an outward-directed pressure to create sufficient space for rounding. This pressure deforms the extracellular confinement (here micropillars) affecting the mitotic cell. The deflection of the micropillars measures the force generated by the rounding mitotic cell. If the confinement cannot be deformed sufficiently to allow the cell to round, the mitotic cell pushes itself out of the confinement where it can conduct mitosis in an unperturbed manner. After mitosis the two daughter cells move back into the confinement. (**b**) Mitotic cells that cannot create sufficient pressure to deform their confinement or to push themselves out of the confinement cannot round properly. Cells that cannot properly round under confined conditions are subject to an increased likelihood of apoptosis.

In this paper, we have engineered micropillar arrays to mimic the mechanical confinement of epithelia and to measure the forces generated by single epithelial cells rounding for mitosis (Fig. 7). The lateral forces generated by rounding MDCK cells progressing through mitosis ranged from 3 to 17 nN (Fig. 3). These rounding forces may be considered relative because they were calculated based on the assumption that the rounding cell more efficiently pushes the upper region of the micropillars to deflect them (Fig. 4; Supplementary Figs 4 and 10–12). If the rounding cell in reality pushes at the lower region of the pillar, the rounding forces would be higher than calculated here. Importantly, assuming that rounding mitotic cells of the same type on average push against approximately the same region of the micropillars, the force measurements here provide relative values. Chemical perturbation experiments showed that, as has been described earlier for mitotic HeLa cells^14^, mitotic MDCK cells also generate a rounding force via the contracting actomyosin cortex and expansive hydrostatic pressure. From confocal microscopy images, we estimated a contact area of 18 × 10^–12^ m^2^ (≈10% of the micropillar surface) between mitotic cell and pillar, consequently the measured rounding forces originate from an outward-directed pressure of ≈170–890 Pa. This pressure range includes that of ≈310 Pa determined by AFM (Supplementary Fig. 7) and is similar to the rounding pressure of ≈400–500 Pa recently determined for mitotic HeLa cells^14^,^15^,^16^.

Remarkably, when using micropillar arrays in which the pillar spacing was too close, cells moved vertically to escape confinement (Fig. 7a). This movement out of confinement was an intrinsic property of the rounding force generated by the mitotic cell. Once free from mechanical confinement, cells conducted mitosis and the daughter cells returned to the space between the confining pillars. Such mechanisms are reminiscent of those observed with pseudostratified epithelial cells in *Drosophila*, the sea anemone *Nematostella vectensis* and neural tube of vertebrates^18^,^47^. In a process termed interkinetic nuclear migration, cells move during mitosis towards the apical surface where they can round in an unperturbed manner. Upon completion of mitosis, daughter cells then return to their columnar shape and reintegrate back into the epithelium^7^,^9^,^18^,^48^. By this mechanism, which ensures the generation of a pseudostratified epithelium, the cell escapes confinement in the epithelium during mitosis^49^,^50^. This suggests that these cellular movements *in vitro* are driven by the intrinsic properties of cells trying to round against confinements that are too stiff.

Our results also show that mitotic cells that can neither round against confinement nor escape this confinement cannot properly orient their spindles (Figs 6 and 7b). As a consequence of their impaired rounding, confined mitotic cells are significantly less viable and more likely to undergo apoptosis (Fig. 5). In epithelia, spindle orientation plays a vital role in ensuring tissue growth and integrity^26^,^30^. Cell rounding is required for planar spindle orientation in some epithelia *in vivo*^18^,^40^. Spindle alignment directs the plane of cytokinesis, and alignment in the plane of the epithelial tissue promotes tissue growth and spreading, whereas perpendicular spindle alignment redirects the plane of cytokinesis. Redirection of the plane of cytokinesis when the spindle aligns perpendicularly to epithelium can lead to defective tissue morphogenesis, because the plane of cytokinesis determines the relative position of daughter cells produced by mitosis. It may be speculated that impaired mitotic rounding and misalignment of the mitotic spindle independently or synergistically influence cellular viability. Moreover, as such defects in spindle orientation are not easy to produce in cultivated epithelial or monolayered cells (Fig. 6), our micropillar assay can be useful to study the molecular mechanisms that regulate mitotic spindle orientation^51^,^52^,^53^,^54^.

The availability of large pillar arrays and the optical readout of the assay enables the simultaneous characterization of hundreds of single cells to investigate the cellular mechanisms that drive the drastic shape change in mitosis. Thus, the micropillar assay opens up the possibility to perform a large-scale screen for genes and mechanisms involved in mitotic cell rounding. We have demonstrated that the contributions of proteins involved in mitotic cell rounding to generating force can be quantified by combining the micropillar assay with targeted chemical perturbation. Mechanical information can be correlated to morphological phenotypes by the simultaneous application of confocal fluorescence microscopy. Finally, the micropillar assay is a versatile tool that can be readily combined with state-of-the-art fluorescence microscopy to study mitotic rounding at high throughput.

## Methods

### MDCK and HeLa cell culture

Wild-type MDCK (clone II, kindly provided by A. Helenius, ETH Zürich) cells were cultured in DMEM supplemented with 2 mm GlutaMAX, 10% fetal bovine serum (FBS), 100 μg ml^–1^ penicillin and 100 μg ml^–1^ streptomycin (all Invitrogen) at 37 °C with 5% CO_2_. To generate an MDCK II cell line stably expressing H2B-eGFP and actin-mCherry, we subcloned sequences encoding for H2B-eGFP and actin-mCherry into the lentiviral vector pRRLsincPPT-hPGK. Using a standardized protocol^55^, we obtained lentiviruses carrying the H2B-eGFP and actin-mCherry transgenes. These lentiviruses were co-transduced to MDCK cells. MDCK cells stably expressing both H2B-eGFP and actin-mCherry were selected by fluorescence-activated cell sorting and propagated in the culture conditions described above. The MDCK II cell line stably expressing E-cadherin-eGFP was kindly provided by W. James Nelson (Stanford University, CA). For selection, H2B-eGFP- and actin-mCherry-expressing MDCK cells were cultured in the presence of 0.5 μg ml^–1^ puromycin (Invitrogen) and E-cadherin-eGFP-expressing MDCK cells were cultured in the presence of 0.1 μg ml^–1^ kanamycin (Invitrogen). For epithelia generation, MDCK cells were seeded (0.8 × 10^6^ cells per well) on trans-wells (0.4 μm pore size, Corning Fisher, NY) and were visualized on day 3 and 14 of growth. To analyse mitotic cell rounding, the cells were plated on micropillar substrates cast on 35-mm-diameter glass-bottom Petri dishes (WPI, Saratosa, FL) and grown for 24 h until they reached ≈50% confluence.

HeLa-Kyoto cells^16^,^56^,^57^ expressing a histone H2B-eGFP construct^56^ and with CAAX-mCherry fluorescently marking the plasma membrane and HeLa-Kyoto cells expressing H2B-mCherry and tubulin-eGFP^16^,^57^ were maintained in DMEM with 2 mM GlutaMAX supplemented with 10% FBS, 100 μg ml^–1^ penicillin, at 37 °C with 5% CO_2_. For selection, H2B-eGFP- and CAAX-mCherry-expressing HeLa-Kyoto cells were cultured in the presence of 0.5 μg ml^–1^ puromycin (Invitrogen), whereas H2B-mCherry- and tubulin-eGFP-expressing HeLa-Kyoto cells were selected in the presence of 0.5 μg ml^–1^ geneticin (Invitrogen) and 0.5 mg ml^–1^ puromycin (Invitrogen).

We determined the following mitotic phases from fluorescence microscopy images of fluorescently (eGFP or mCherry) labelled histones: prophase, condensed chromosomes but intact nucleus; prometaphase, nuclear envelope breakdown; metaphase, chromosomes aligned to form a metaphase plate; anaphase, two sets of chromosomes separated.

### Perturbants

Chemical perturbants were acquired from Sigma-Aldrich, except SMHIF2 (Merck), calpeptin (Cytoskeleton), IPA3 and CK666 (Tocris). Perturbants were used at the indicated concentrations. A microsyringe (Hamilton) was used to inject the perturbants into the Petri dish containing the micropillar substrates. Supplementary Table 1 lists the perturbants used, how they were applied (preincubated or added after nuclear envelope breakdown) and a short description of their action.

### Pattern formation and chemical preparation of micropillars

Micropillar arrays were formed through replication moulding with a Si master, which was produced by photolithography and deep reactive-ion etching processes (Supplementary Fig. 2). Briefly, the desired micropillar pattern was transferred to a positive photoresist on a silicon substrate by photolithography. After development and dissolution of the exposed photoresist, holes were etched into the Si substrate to the desired depth using deep reactive-ion etching. After removal of the photoresist and cleaning, the wafers were passivated with a silanization agent (tridecafluoro-1,1,2,2-tetrahydrooctyl trichlorosilane, ABCR, Karlsruhe, Germany) in a vacuum desiccator to facilitate separation of the elastomer from the wafer. A liquid silicone pre-polymer, PDMS (Sylgard184, Dow Corning) was then poured over the silicon template, cured at 65 °C for 6 h and then peeled off. The lengths, conical shape and spacing of micropillars were varied. We used hexagonal arrangements of conical micropillars with interpillar distance of ≈6.8 μm and pillar length of ≈8.2 μm, interpillar distance of ≈8.8 μm and pillar length of ≈9.2 μm and interpillar distance of ≈14.3 μm and pillar length of ≈13.7 μm. After release from the mould, the PDMS replica was oxidized and sterilized in oxygen plasma for 2 min. This process rendered the PDMS surface hydrophilic, which was required to facilitate adsorption of fibronectin (incubation for 3 h in 50 μg ml^–1^ fibronectin (Sigma-Aldrich) at 37 °C in PBS buffer). To analyse the micropillar positions, the pillars were fluorescently stained by incubation for 3 h with 20 μg ml^–1^ lipophilic carbocyanine dye DiD (1,1′-dioctadecyl-3,3,3′3′-tetramethylindocarbocyanine, 4-chlorobenzenesulfonate salt, Invitrogen).

### Cell viability and proliferation assay

The viability of MDCK cells cultured on micropillars in the presence of different chemical compounds was studied using a 3-(4,5-dimethylthiazol-2-yl)-2,5-diphenyltetrazolium bromide (MTT, Sigma-Aldrich Chemicals) assay^58^. Briefly, 1 ml of culture medium containing 0.5 μg ml^–1^ MTT was added to each micropillar device, followed by incubation at 37 °C for 3 h. The optical density was measured at 490 nm (Multiskan MK3, Thermo Labsystems). The percentage cell viability was calculated through the following equation:


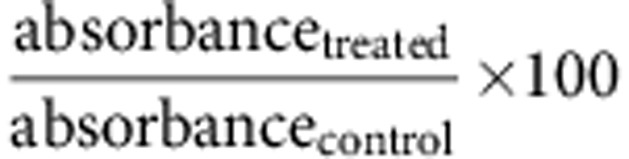


Cell viability was expressed as mean±s.d. Differences in cell proliferation between cells treated with chemical compounds and untreated control cells were tested for by Mann–Whitney tests. *P* values <0.05 were considered statistically significant. To distinguish between apoptotic and living cells, cells grown on flat surfaces (for example, cell culture plates) or on micropillar devices were first perturbed with the specific molecular compounds as described. After this, the cells were stained for 30 min with annexin V-Cy3 and 6-carboxyfluorescein diacetate (APOAC apoptosis detection kit, Sigma-Aldrich Chemicals). According to the manufacturers' instructions, annexin-V-positive cells were identified as apoptotic, and cells positive for 6-carboxyfluorescein diacetate were defined as live using fluorescence microscopy. Cell numbers were automatically determined through analysis of the fluorescence confocal microcopy images using CellProfiler 2.0 (BROAD Institute, Massachusetts, USA).

### Time-lapse confocal microscopy

Living cells were observed using an inverted confocal microscope (Nikon TiE equipped with an A1R confocal laser scan head, Nikon, Switzerland). The set-up was equipped with an environmental chamber (Life Imaging Services, Basel, Switzerland) to maintain 37 °C at relative humidity of 90 and 5% CO_2_ in air during data acquisition. Mitotic cell rounding was analysed using a Nikon Plan Fluor 40 × 0.95 numerical aperture long-distance air objective at 15 min intervals for up to 12 h. Transmission and fluorescence images of cells and micropillars were acquired using a transmitted light channel and filter settings for mCherry, eGFP and DiD, at each time point. Confocal *z*-stacks of the cells growing between micropillars were collected every 1.5 μm over a range of 30 μm starting at the bottom of the pillars and ending above the pillars. The orientation of the mitotic spindle relative to the plane of the substrate was determined in metaphase cells by first measuring the angle of the fluorescently labelled chromosomes aligned along the metaphase plate. The height of the metaphase spindle was measured relative to the substrate plane as indicated (Fig. 6c).

### Measuring the maximum cell height during metaphase

We measured the maximum cell height during metaphase using the sectioning toolbar of Imaris software (Bitplane). For each time interval during mitosis, an *x*–, *y*–, *z*– confocal section was analysed to determine the position of cells between the micropillars. Since cells protruded from the pillars during metaphase, we acquired a 30-μm-thick confocal section at each time interval. The first step of height measurement consisted of a manual assignment of the base of the micropillar device guided by the DiD fluorescence signal. The top of the cell was defined by reference to the actomyosin cortex (actin-mCherry), while the cell state was monitored using the eGFP-labelled histone. The height of metaphase cells was measured from *x*–, *z*– projections. Differences in height between metaphase cells treated with chemical compounds and untreated controls were considered statistically significant when the two-tailed Mann–Whitney *U*-test resulted in a *P* value <0.05.

### Scanning electron microscopy (SEM)

For SEM (Nova Nano SEM230, FEI) imaging micropillar substrates were dehydrated by rinsing with graded ethanol/water mixtures (50, 70, 80, 90 and 100%, each step for 10 min at 4 °C). Ethanol was slowly exchanged successively by amyl acetate and liquid CO_2_. Finally, samples were dried using the critical point method (CPD 030, Balzer) and then sputter-coated with a ≈20-nm-thick layer of gold.

### Trans-mitotic constant height assay

H2B-eGFP- and actin-mCherry-expressing MDCK cells were seeded on glass-bottom Petri dishes (WPI) coated with Matrigel (Matrigel Basement Membrane Matrix, BD) and cultured as described above. Before experiments, medium was changed to DMEM (high glucose, pyruvate, Life Technologies) with 4 mM NaHCO_3_ (Sigma-Aldrich) buffered with 20 mM HEPES (AppliChem) at pH 7.4 supplemented with 10% (vol/vol) FBS, 100 μg ml^–1^ penicillin and 100 μg ml^–1^ streptomycin. All microscopy equipment was placed in and all experiments were carried out in a custom-made temperature-stabilized box at 37 °C (The Cube, Life Imaging Services). An AFM (Cell Hesion 200, JPK Instruments) was mounted on an inverted optical microscope (Cell Observer.Z1, Zeiss). Wedged AFM cantilevers^57^ calibrated using the thermal noise method showed average spring constants of ≈0.8 N m^–1^. Cantilever sensitivity was determined against immobilized polystyrene beads as described^59^,^60^. The height of the support adjacent to the target cell was determined and taken as a reference. The cantilever end was then positioned over the cell, 10 μm above the substrate. Prophase cells typically had heights of <8 μm, and were therefore not initially in contact with the cantilever. During rounding, the cell made contact with the wedged cantilever, and the cantilever deflection and the corresponding force were recorded over time. Simultaneously, differential interference contrast (DIC) and GFP fluorescence images at the mid-plane of the cell were recorded and the horizontal cross-sectional area of the cell was determined using the optical microscope software (AxioVision, Zeiss). Rounding pressure was derived by dividing the rounding force by the contact area of the cell and cantilever, which was estimated from the measured horizontal cross-sectional area based on the assumption of a semicircular profile^15^.

### Characterization of the micropillar spring constant

To evaluate the Young's modulus of the PDMS, stress–strain experiments were carried out at room temperature with a tensile machine (Instron model 5542). PDMS rectangular arrays of 26 mm in perimeter were tested at an initial strain rate of 2 mm min^–1^. The slope at the start of the deformation was used to calculate the Young's modulus *E*. We found that by curing PDMS at 65 °C for 6 h, the *E* was 3±0.3 MPa. After measuring the micropillar dimensions by SEM, we calculated micropillar spring constants according to:


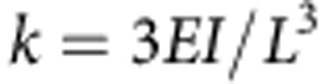


where *I* is the moment of inertia and *L* the length of the micropillar^61^. Alternatively, we used an AFM (Nanowizard II, JPK Instruments) to directly evaluate the spring constant of shorter micropillars with lengths of 8.2 μm and of longer micropillars with lengths of 13.7 μm (Supplementary Fig. 2). To do this, the top of a micropillar was brought into contact with an AFM cantilever. The AFM cantilever was used to apply a force to a micropillar and the micropillar deflection was measured. From this deflection, we determined the average spring constant of the shorter micropillars.

### Finite-element method analysis of micropillar deflection

A commercial finite-element package ANSYS (ANSYS Inc., Southpointe, PA) was used to analyse the deflection of PDMS micropillars exposed to different perpendicular forces. The force was applied to an area of 9.97 × 10^–13^ m^2^ at the upper part of the micropillars (Supplementary Fig. 4a). PDMS micropillars were modelled as neoHookian hyperelastic cylinders with a Young's modulus *E* of 3 MPa and discretized into hexahedral mesh elements. The bottom surface of the micropillars was assigned to fixed boundary conditions. A horizontal force *F* was then applied uniformly at all of the nodes along the surface of the micropillar. Finite-element method analysis was performed to determine displacement (that is, deflection) *δ* of the pillar upon the application of a range of different forces ranging from 8 to 160 nN (Supplementary Fig. 4).

### Measuring forces generated by cells rounding for mitosis

The force generated by cells rounding for mitosis was measured by analysing the deflection of micopillars surrounding the cell. For analysis we used a Matlab code developed for the analysis of pillar deflection kindly provided by Christopher Chen^62^,^63^. Briefly, micropillar arrays and cells were imaged using confocal microscopy. For each cell, fluorescence images of the DiD-stained PDMS micropillars were acquired at two different focal planes. One image was taken at the top plane of the micropillars and the other image was taken ≈1 μm above the PDMS base. These two fluorescence images were analysed with the custom Matlab code to calculate the deflection of the micropillar and thus the force generated by the rounding mitotic cell. For a description of the algorithm steps, see Supplementary Fig. 5.

### Statistical analysis

Averages and mean values are given with±s.e.m. The number of single cells analysed has been specified for each condition (see the figures and legends). Statistical analyses were made using the two-tailed Mann–Whitney *U*-test with *P* values ≥0.05 not being considered significant. Significant *P* values were categorized into values <0.05, <0.01 and <0.001.

## Additional information

**How to cite this article:** Sorce, B. *et al.* Mitotic cells contract actomyosin cortex and generate pressure to round against or escape epithelial confinement. *Nat. Commun.* 6:8872 doi: 10.1038/ncomms9872 (2015).

## Supplementary Material

Supplementary Information Supplementary Figures 1-12, Supplementary Table 1 and Supplementary References
