# Supplementary material for: Mitotic cells contract actomyosin cortex and generate pressure to round against or escape epithelial confinement
Source: Nat Commun. 2015 Nov 25;6:8872. doi: 10.1038/ncomms9872 (PMC4696517; doi:10.1038/ncomms9872)
Supplement: Supplementary Information — Supplementary Figures 1-12, Supplementary Table 1 and Supplementary References [file ncomms9872-s1.pdf]

SUPPLEMENTARY INFORMATION

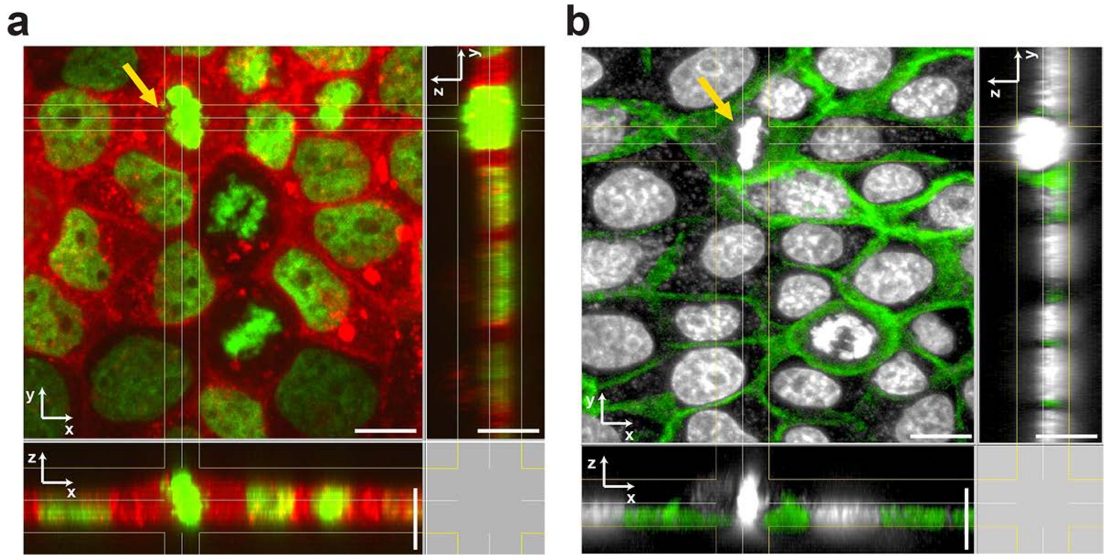

Supplementary Figure 1. Mitosis in polarized MDCK cells after 14 days of culture. (**a**) Confocal microscopy image of polarized MDCK cells expressing actin-mCherry and H2B-eGFP showing one mitotic cell rounded up in metaphase (yellow arrow). (**b**) Confocal microscopy image of MDCK cells expressing E-cadherin-eGFP and showing extensive contacts between metaphase (yellow arrow) and interphase cells. MDCK cells expressing (**a**) actin-mCherry (red) and H2B-eGFP (green) and (**b**) E-cadherin-eGFP (green) and nuclei stained with Hoechst 33342 (grey) were plated on permeable filter supports and grown for 14 days before data acquisition. Scale bars, 10 μm.

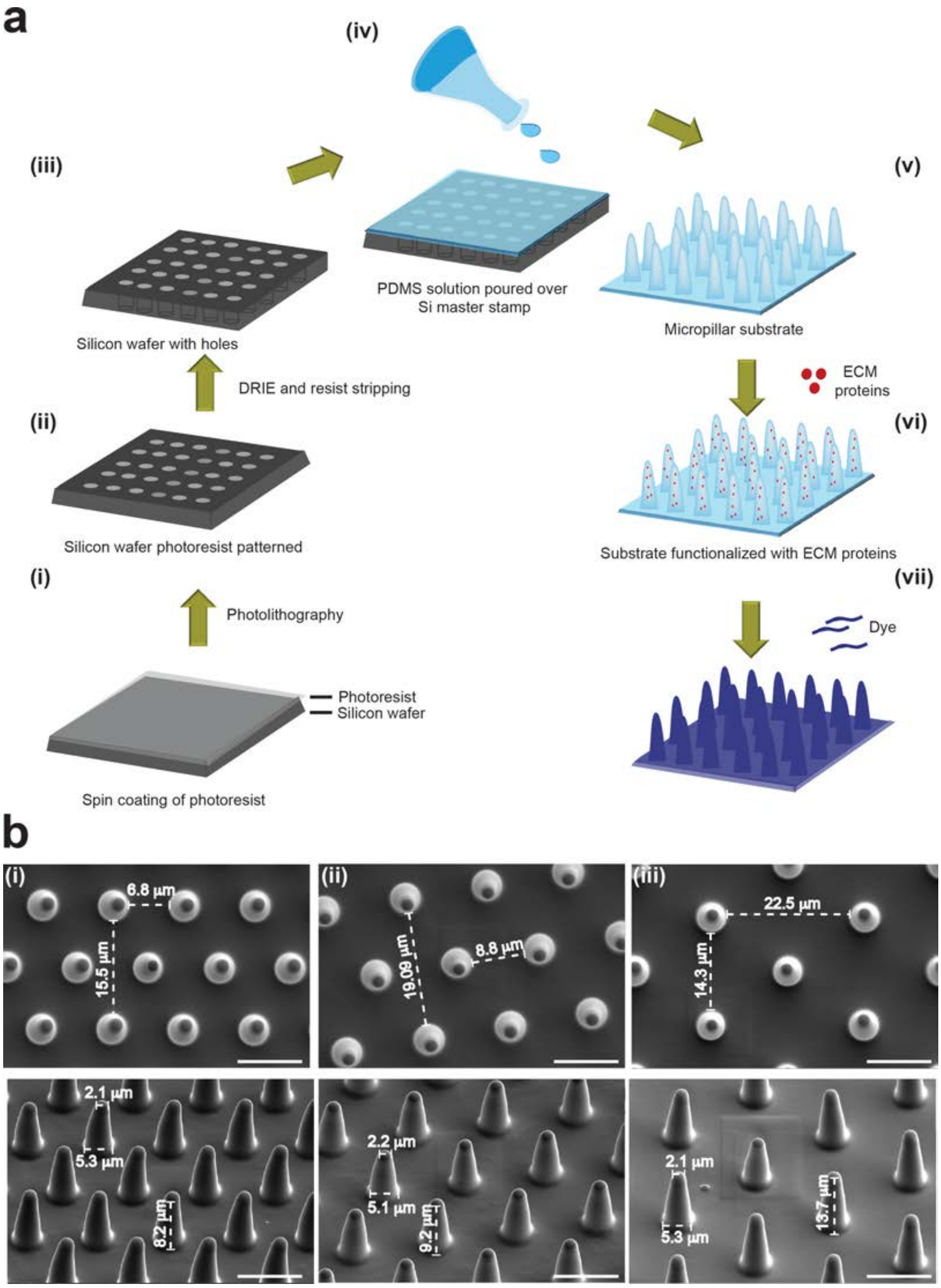

Supplementary Figure 2. Production and characterization of PDMS micropillar arrays. (**a**) Illustration of the steps used to prepare micropillar arrays. The silicon master with holes is produced by (i) spin coating the photoresist on the silicon wafer followed, by (ii) photolithography, deep reactive-ion etching (DRIE), and (iii) resist stripping. (iv) Then, the PDMS solution is poured on the silicon wafer, which had just prior to this been treated in oxygen plasma for 30 s. (v) After this, the PDMS substrate is peeled from the silicon wafer and features large arrays of micropillars. These PDMS micropillars were functionalized with (vi) fibronectin and (vii) fluorescent dye. (**b**) Scanning electron microscopy images of micropillar arrays used in our studies. Pillars were conical being ≈5 μm wide at the bottom and ≈2 μm wide at the top. Micropillars (i) spaced 6.8 ± 0.1 μm (average ± S.D., n = 123) and featuring lengths of 8.2 ± 0.2 μm, (ii) spaced 8.8 ± 0.2 μm and featuring lengths of 9.2 ± 0.2 μm (n = 95), and (iii) spaced 14.3 ± 0.2 μm and featuring lengths of 13.7 ± 0.2 μm (n = 80). Distances between pillars were measured from bottom rims. Scale bars, 10 μm.

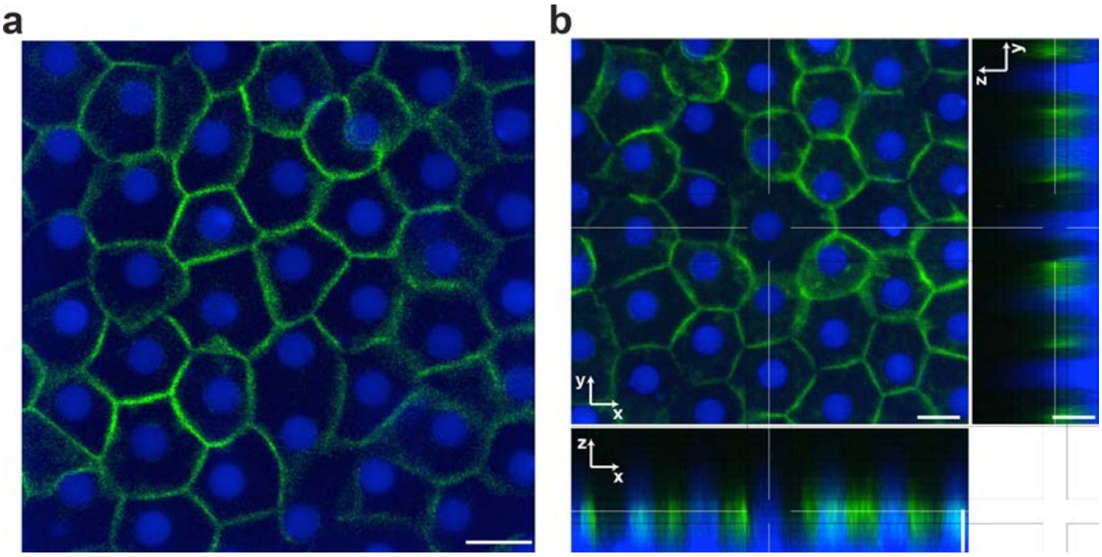

Supplementary Figure 3. Polarized MDCK cells cultured for 14 days on a micropillar array. (**a**) Confocal microscopy image of polarized MDCK cells expressing E-cadherin-eGFP (green) grown on a DiD labeled micropillar array (blue) having an average pillar spacing of ≈14.3 μm and pillar length of ≈13.7 μm. Scale bar, 10 μm. (**b**) Confocal microscopy image of MDCK cells expressing E-cadherin-eGFP and showing extensive tight junction contacts between the cells. MDCK cells were plated on DiD labeled micropillar arrays and grown for 14 days before data acquisition. Lateral scale bar, 8 μm and vertical scale bars, 10 μm.

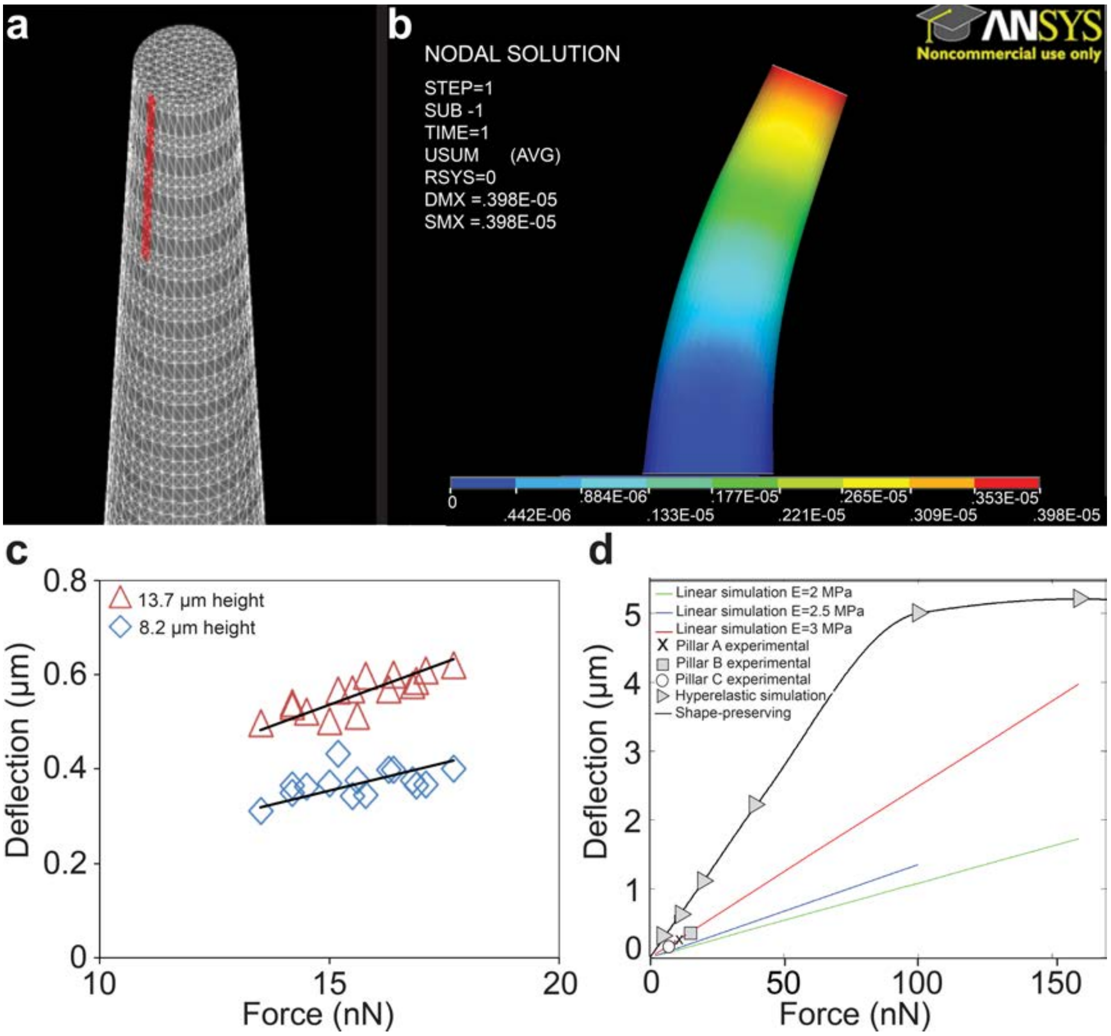

Supplementary Figure 4. Micropillar deflection simulated using finite-element analysis (FEA) and experimentally determined using an AFM cantilever. (**a**) Three-dimensional grid of finite elements of a micropillar used to simulate the micropillar deflection. The force was applied to a 9.97×10^{-13} m^2 large area at the upper part of the micropillar (highlighted red). (**b**) FEA simulation illustrating the deflection of the micropillar exposed to a force of 70 nN. (**c**) Deflection of single micropillars with lengths of 8.2 μm and 13.7 μm measured as a function of the applied force. Data points represent the deflection of individual micropillars to which a defined force has been applied using a reference AFM cantilever. The lines represent fits used to determine the spring constants of the 13.7 μm long (29.0 ± 2.6 mN m^{-1}; mean ± S.D., n = 30) and 8.2 μm short (42.9 ± 4.0 mN m^{-1}, n = 30) micropillars. (**d**) Hyperelastic and linear simulations using different Young's moduli E. The superimposed experimental data points agree with the linear model at E = 3 MPa (red line) and with results reported[1].

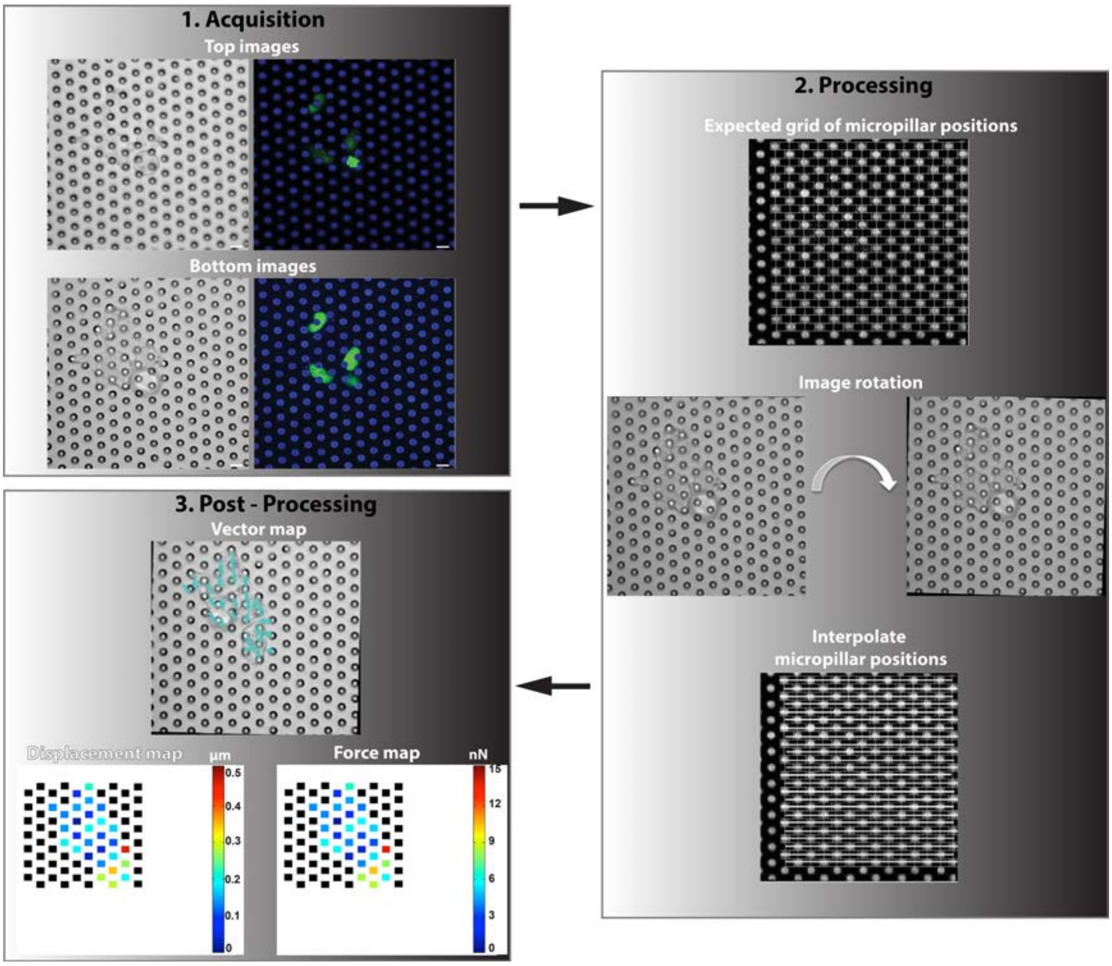

Supplementary Figure 5. Analyzing micropillar deflection and force generated by mitotic cells. Key steps for analyzing the micropillar deflection and force generated by a cell are illustrated the following way: Step 1: Acquisition confocal microscopy images of cells on micropillars by using a 40x objective. Confocal images were taken at the bottom and top of the micropillars. Step 2: Analyzing micropillar positions. Overlaid images are then displayed; a grid is superimposed to define the micropillar positions. Then, the top and bottom images are rotated to correct translational shifts. After this, the images are analyzed by using the algorithm for spot detection and centroid determination. The resulting centroids are then sorted into two matrices containing the centroid for each pillar in the top and the base images. The displacements are then calculated by subtracting the base image centroids from the top image centroids. Step 3: Quantifying the micropillar deflection and conversion of the deflection to force. The data derived from step 2 are exported to calculate a vector map (blue arrows) characterizing the micropillar deflection. From this vector map a deflection map and a force map are calculated. The custom-written MATLAB algorithm was kindly provided by Christopher Chen (University of Pennsylvania)[2].

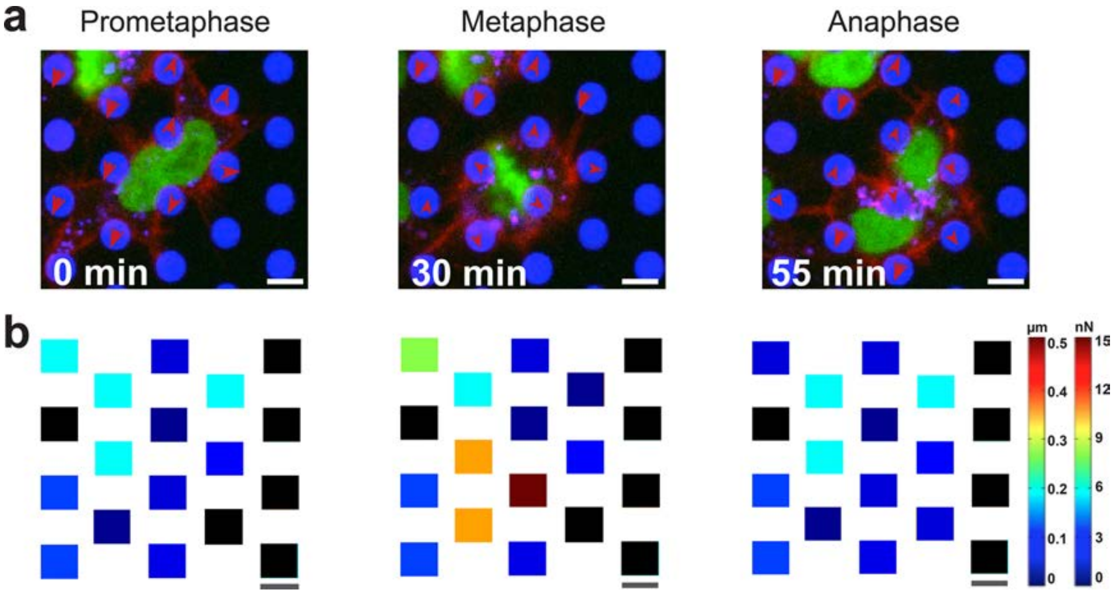

Supplementary Figure 6. Micropillar deflection and force generated by rounding mitotic HeLa cells. (**a**) Confocal microscopy images of HeLa cells expressing H2B-eGFP (green) and actin-mCherry (red) grown between DiD labeled micropillar arrays (blue) with an average spacing of ≈14.3 µm and length of ≈13.7 µm (Supplementary Fig. 2). When entering mitosis the rounding HeLa cells deflect adjacent micropillars (red arrows) until cell division has been conducted. Shown are HeLa cells progressing from prometaphase to metaphase and anaphase. (**b**) Displacement and force maps of HeLa cells recorded in prometaphase, metaphase and anaphase. Scale bars, 8 µm.

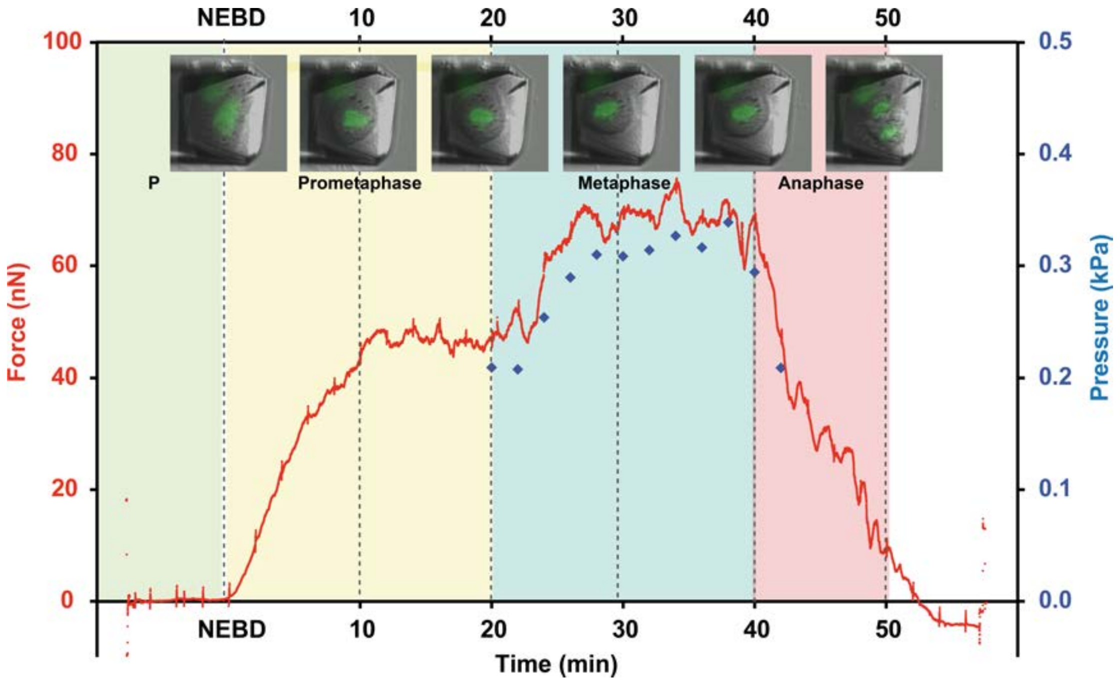

Supplementary Figure 7. Trans-mitotic constant height assay to quantify the force and pressure generated by a MDCK cell rounding in mitosis. Overlaid differential interference contrast (DIC) and histone H2B-eGFP (green) fluorescence images of a single mitotic MDCK cell at time points indicated above. Shown is the measured force (red) and calculated pressure (blue) generated by the rounding MDCK cell (see Methods). To quantify the force of cell rounding a tipless cantilever was positioned over a prophase MDCK cell, at a height of 10 μm above the substrate[3,4]. When becoming round in prometaphase, the mitotic cell pushes against the confining AFM cantilever. Rounding pressure was derived by dividing the rounding force of a mitotic cell by the contact area of the cell with the cantilever. This contact area was estimated from the measured horizontal cross-sectional area based on the assumption of a semi-circle shape profile. Three independent MDCK cells revealed an average force of 43 ± 18 nN (average ± S.D.) and pressure of 310 ± 35 Pa (average ± S.D.) generated in metaphase. Time zero denotes nuclear envelope breakdown (NEBD). Mitotic phases highlighted are: prophase (P, green), prometaphase (yellow), metaphase (blue) and anaphase (pink).

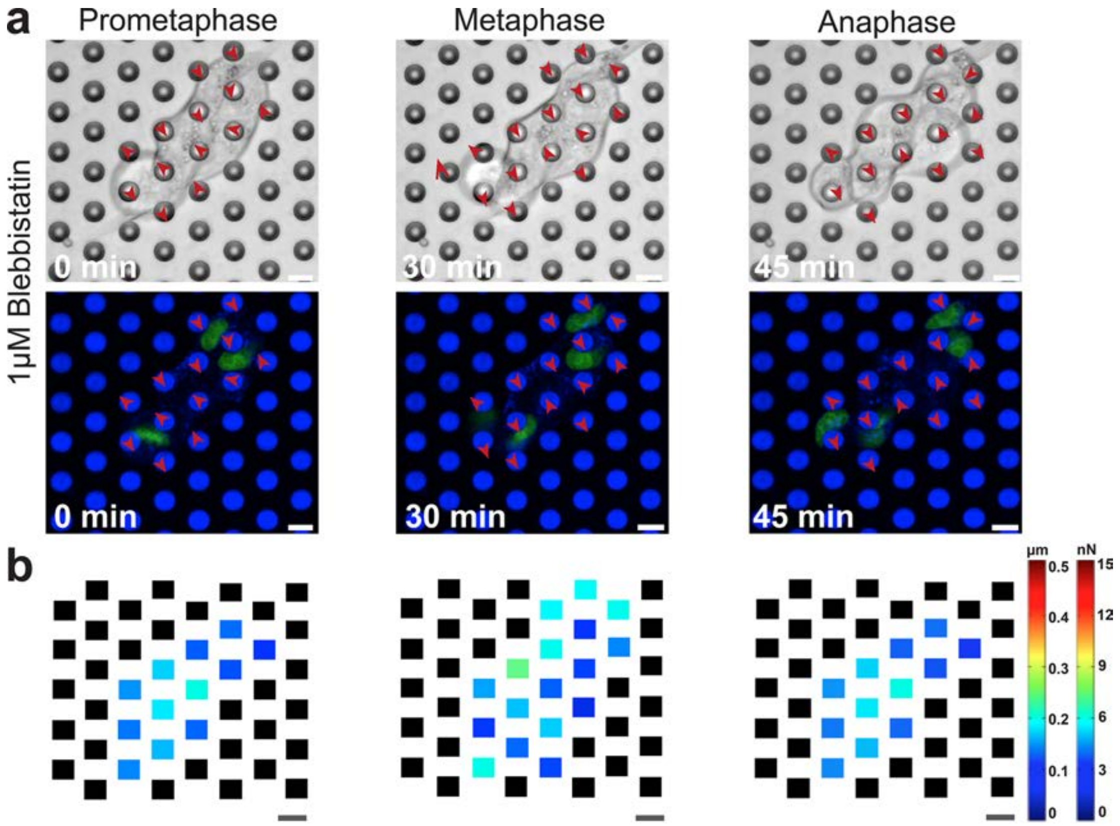

Supplementary Figure 8. Micropillar deflection and force generated by rounding mitotic MDCK cells in the presence of blebbistatin. (**a**) Differential interference contrast (DIC, top row) and confocal (bottom row) images of MDCK cells expressing H2B-eGFP grown on micropillars with an average distance of ≈14.3 μm and length of ≈13.7 μm (Supplementary Fig. 2) for 2 h in the presence of 1 μM blebbistatin. MDCK cells were histone H2B-eGFP labeled (green) and micropillars DiD labeled (blue). (**b**) Displacement and force maps of MDCK cells recorded in prometaphase, metaphase and anaphase in presence of 1 μM blebbistatin. Scale bars, 10 μm.

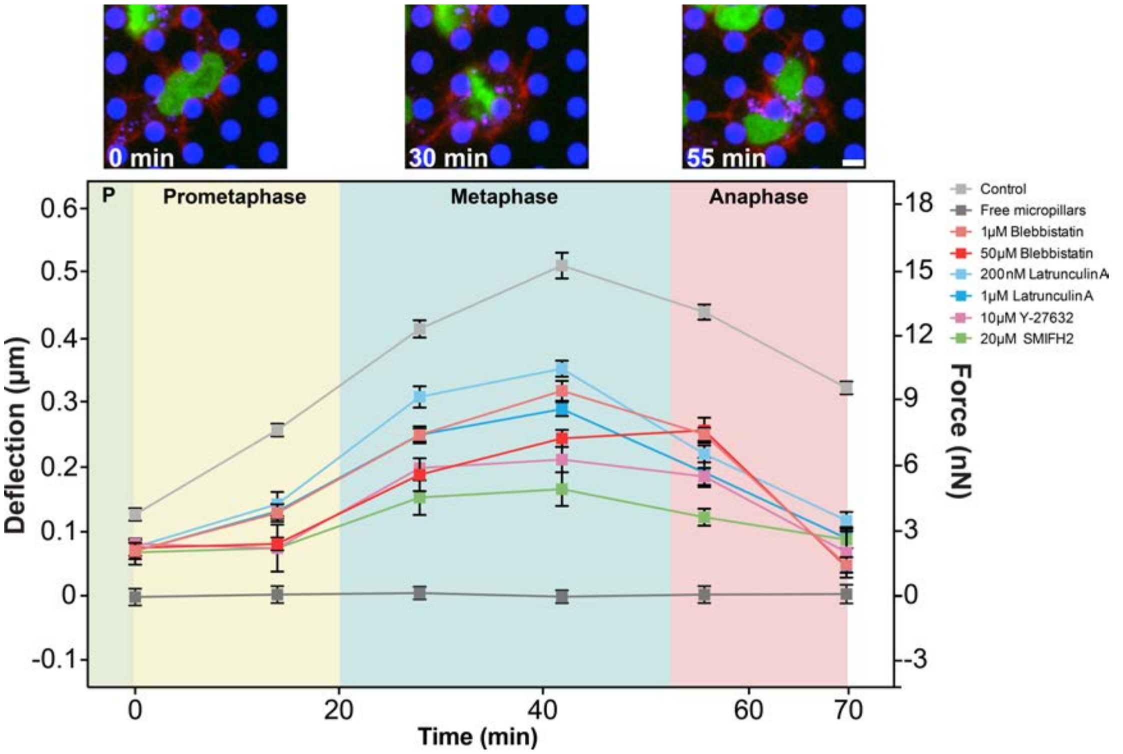

Supplementary Figure 9. Forces generated by HeLa cells progressing through mitosis and confined by micropillars. Micropillar deflection and force generated by rounding mitotic HeLa cells increase from prophase to prometaphase until they reach maximum values in metaphase. In anaphase the micropillar deflection and thus the rounding force drop significantly. The generation of rounding force by mitotic HeLa cells was perturbed using different chemical compounds. 'Control' indicates pillar deflection measured in absence of any chemical perturbation and 'free micropillars' indicates pillar deflection in absence of cells (i.e. experimental noise). Fluorescence images show overlaid signals recorded from eGFP-labeled histone (H2B-eGFP, green), CAAX-mCherry (red) and DiD labeled micropillars (blue) at the times indicated. Time zero denotes the nuclear envelope breakdown. Background of the graph is colored to indicate the time range in which an example cell progresses through prophase (P, green), prometaphase (yellow), metaphase (blue) and anaphase (pink). Micropillars had an average distance of 14.3 µm and length of 13.7 µm. For each condition the average value and standard error of the mean (s.e.m., error bars) of n = 45 cells are given. Scale bar, 6 µm. For a description of the perturbants see Supplementary Table 1.

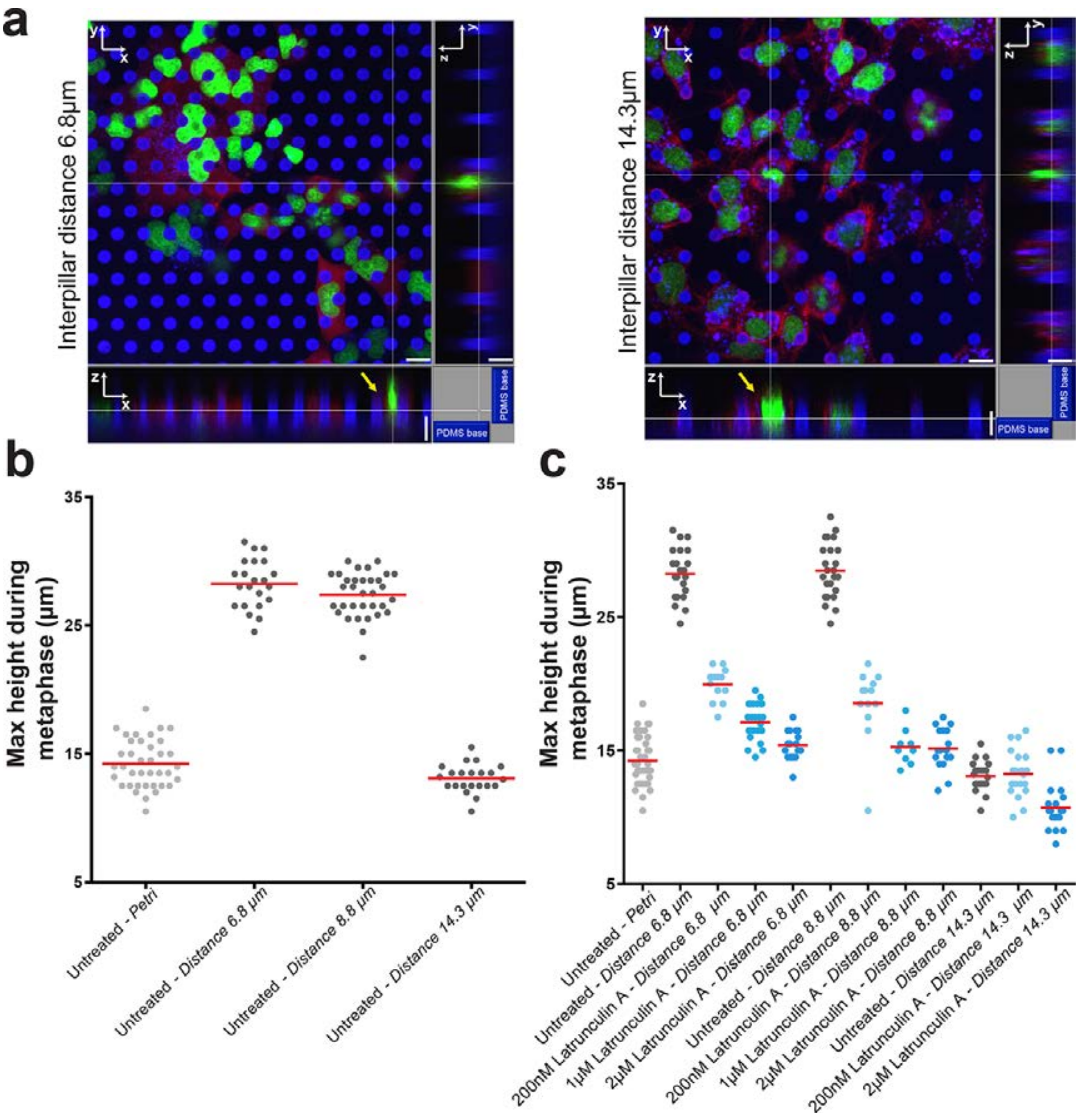

Supplementary Figure 10. Height elevation of mitotic MDCK cells confined by micropillars. (**a**) Confocal microscopy images of MDCK cells grown on micropillar arrays with interpillar distances of 6.8 µm (*left*) and 14.3 µm (*right*). MDCK cells expressed CAAX-mCherry (red) and H2B-eGFP (green). The image shows that MDCK cells constrained by interpillar distances of 6.8 µm move out of the DiD labeled micropillar array (blue) when going through mitosis (indicated by yellow arrow in left image). In contrast, mitotic MDCK cells grown on micropillar arrays with interpillar distances of 14.3 µm remain between the pillars (indicated by yellow arrow in right image). (**b**) Maximum height from the bottom of the substrate to the top of rounding mitotic cells reached in metaphase. Cells have been grown on micropillar arrays with interpillar distances of 6.8 µm, 8.8 µm, or 14.3 µm. (**c**) Characterizing the effect of latrunculin A on the maximum height that mitotic cells reach in metaphase on micropillar arrays with interpillar distances of 6.8 µm, 8.8 µm, or 14.3 µm. Each dot represents one experimentally characterized cell. For each condition the bar denotes the average. Scale bars, 8 µm (left image) and 10 µm (right image).

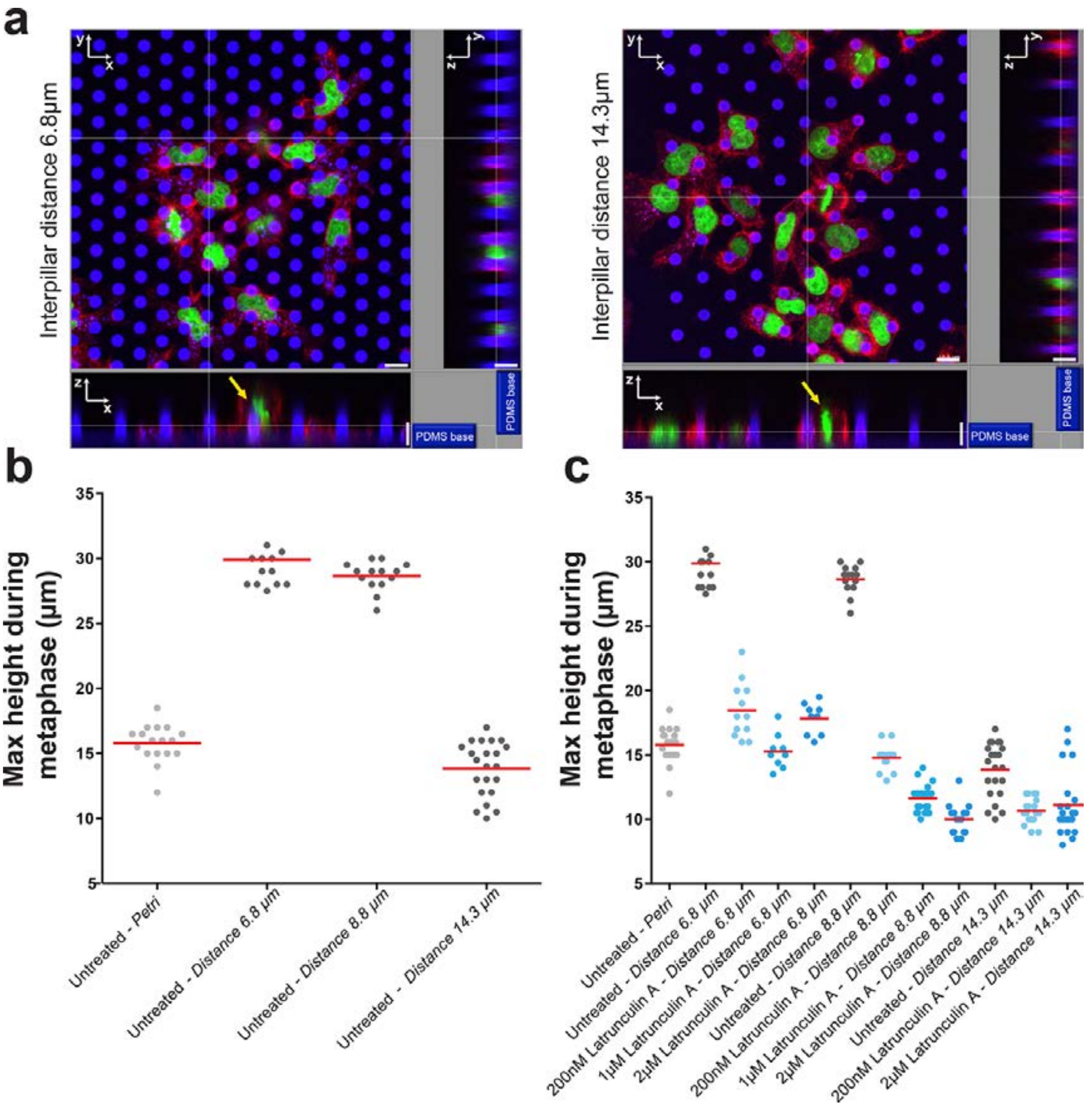

Supplementary Figure 11. Height elevation of mitotic HeLa cells confined by micropillars. (**a**) Confocal microscopy images of HeLa cells grown on micropillar arrays with interpillar distances of 6.8 μm (*left*) and 14.3 μm (*right*). HeLa cells expressed CAAX-mCherry (red) and H2B-eGFP (green). The images show that mitotic HeLa cells move out of the DiD labeled micropillar array (blue) when constrained by interpillar distances of 6.8 μm (as indicated by yellow arrow, *left*). In contrast, mitotic HeLa cells grown on micropillar arrays with interpillar distances of 14.3 μm remain between the pillars (indicated by yellow arrow in right image). (**b**) Maximum height of the rounding mitotic cells reached in metaphase. Cells were grown on micropillar arrays with interpillar distances of 6.8 μm, 8.8 μm, or 14.3 μm. (**c**) Characterizing the effect of latrunculin A on the maximum height mitotic cells reach in metaphase on micropillar arrays with interpillar distances of 6.8 μm, 8.8 μm, or 14.3 μm. Each dot represents one experimentally characterized cell. For each condition the bar denotes the average. Scale bars, 6 μm (**a**) and 10 μm (**b**).

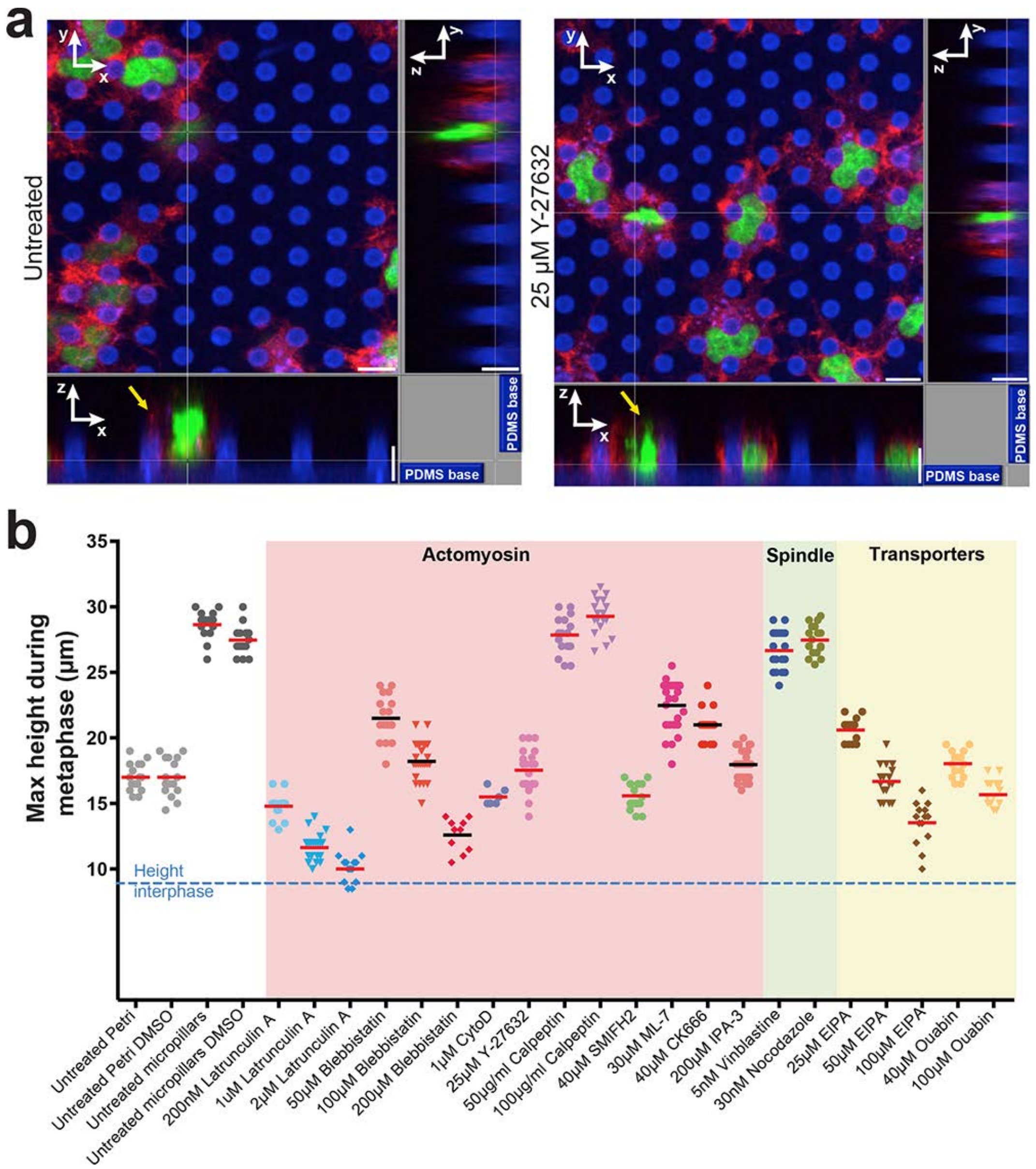

Supplementary Figure 12. Characterizing chemical compounds that perturb the height elevation of mitotic HeLa cells confined by micropillars. (**a**) Confocal microscopy images of HeLa cells expressing CAAX-mCherry (red) and H2B-eGFP (green) grown on DiD labeled micropillar substrates (blue). Micropillars had an average distance of ≈6.8 µm. The left image shows untreated HeLa cells and the right image HeLa cells incubated with 25 µM Y-27632. Yellow arrows indicate mitotic HeLa cells in metaphase. Scale bars, 6 µm. (**b**) Screening of chemical compounds that influence the height mitotic HeLa cells can reach to escape their mechanical confinement. Compounds are categorized into those that effect actomyosin (pink background), the microtubule spindle (green background), and transporters (orange background). All drugs were added 20 minutes before cells entered prophase except for EIPA and Ouabain, which were added during prophase because they inhibit G2/M transition. Maximum heights from the substrate to the top of metaphase cells as recorded using confocal fluorescence microscopy (see Methods). The maximum heights of non-perturbed interphase HeLa cells on the same micropillar substrates are taken as references (dashed blue line). Each dot represents one experimentally characterized cell. For each condition the bar denotes the average.

Perturbant	Method	Target/Action	Figures	Ref
Latrunculin A	Preincubation	Actin monomer sequester	Figures 3, 4, 5, 6 Supplementary Figures 8, 9, 10, 11	5
Blebbistatin	Preincubation	Myosin II inhibitor	Figures 3, 4, 5, 6 Supplementary Figures 7, 8, 11	6
Y-27632	Preincubation	Rho Kinase inhibitor	Figures 3, 4, 5, 6 Supplementary Figures 8, 11	7
Calpeptin	Preincubation	RhoA activator	Figure 4 Supplementary Figure 11	8
SMIFH2	Preincubation	Formin inhibitor	Figures 3, 4 Supplementary Figures 8, 11	9
ML-7	Preincubation	MLC Kinase inhibitor	Figure 4 Supplementary Figure 11	10
CK666	Preincubation	Arp 2/3 inhibitor	Figure 4 Supplementary Figure 11	11
IPA-3	Preincubation	Pak1 inhibitor	Figure 4 Supplementary Figure 11	12
Nocodazole	Preincubation	Microtubule polymerization inhibitor	Figures 3, 4 Supplementary Figure 11	13
Vinblastine	Preincubation	Microtubule polymerization inhibitor	Figures 3, 4 Supplementary Figure 11	14
EIPA	Addition-NEBD	Na^+/H^+ antiporter inhibitor	Figures 3, 4 Supplementary Figure 11	15
Ouabin	Addition-NEBD	Na^+/K^+ ATPase inhibitor	Figure 4 Supplementary Figure 11	16

Supplementary Table 1. List of perturbants used. Along with each perturbant is listed the method of introduction to the cell (Method), a concise description of the perturbation (Target/Action), the experiments used (Figures), and a reference (Ref) which describes the use or the mechanism of each perturbant. Perturbants are categorized into those that effect actomyosin (pink), the microtubule spindle (green), and transporters (orange).

Supplementary References

1 Yang, M. T., Reich, D. H. & Chen, C. S. Measurement and analysis of traction force dynamics in response to vasoactive agonists. *Integr Biol-Uk* **3**, 663-674, doi:Doi 10.1039/C0ib00156b (2011).

2 Lemmon, C. A. *et al.* Shear force at the cell-matrix interface: enhanced analysis for microfabricated post array detectors. *Mech Chem Biosyst* **2**, 1-16 (2005).

3 Stewart, M. P. *et al.* Hydrostatic pressure and the actomyosin cortex drive mitotic cell rounding. *Nature* **469**, 226-230, doi:Doi 10.1038/Nature09642 (2011).

4 Stewart, M. P., Toyoda, Y., Hyman, A. A. & Muller, D. J. Tracking mechanics and volume of globular cells with atomic force microscopy using a constant-height clamp. *Nat Protoc* **7**, 143-154, doi:Doi 10.1038/Nprot.2011.434 (2012).

5 Spector, I., Shochet, N. R., Kashman, Y. & Groweiss, A. Latrunculins - novel marine toxins that disrupt microfilament organization in cultured-cells. *Science* **219**, 493-495, doi:Doi 10.1126/Science.6681676 (1983).

6 Straight, A. F. *et al.* Dissecting temporal and spatial control of cytokinesis with a myosin II inhibitor. *Science* **299**, 1743-1747, doi:Doi 10.1126/Science.1081412 (2003).

7 Uehata, M. *et al.* Calcium sensitization of smooth muscle mediated by a Rho-associated protein kinase in hypertension. *Nature* **389**, 990-994, doi:Doi 10.1038/40187 (1997).

8 Schoenwaelder, S. M. & Burridge, K. Evidence for a calpeptin-sensitive protein-tyrosine phosphatase upstream of the small GTPase Rho - A novel role for the calpain inhibitor calpeptin in the inhibition of protein-tyrosine phosphatases. *J Biol Chem* **274**, 14359-14367, doi:Doi 10.1074/Jbc.274.20.14359 (1999).

9 Rizvi, S. A. *et al.* Identification and characterization of a small molecule inhibitor of formin-mediated actin assembly. *Chem Biol* **16**, 1158-1168, doi:Doi 10.1016/J.Chembiol.2009.10.006 (2009).

10 Makishima, M. *et al.* Induction of differentiation of human leukemia-cells by inhibitors of myosin light chain kinase. *Febs Lett* **287**, 175-177, doi:Doi 10.1016/0014-5793(91)80044-4 (1991).

11 Nolen, B. J. *et al.* Characterization of two classes of small molecule inhibitors of Arp2/3 complex. *Nature* **460**, 1031-U1121, doi:Doi 10.1038/Nature08231 (2009).

12 Deacon, S. W. *et al.* An isoform-selective, small-molecule inhibitor targets the autoregulatory mechanism of p21-activated kinase. *Chem Biol* **15**, 322-331, doi:Doi 10.1016/J.Chembiol.2008.03.005 (2008).

13 Debrabander, M. J., Vandeveire, R. M. L., Aerts, F. E. M., Borgers, M. & Janssen, P. A. J. Effects of Methyl [5-(2-Thienylcarbonyl)-1h-Benzimidazol-2-Yl]Carbamate, (R-17934-Nsc-238159), a new synthetic antitumoral drug interfering with microtubules, on mammalian-cells cultured in vitro. *Cancer Res* **36**, 905-916 (1976).

14 Malawist,S.E., Sato, H. & Bensch, K. G. Vinblastine and griseofulvin reversibly disrupt living mitotic spindle. *Science* **160**, 770-772, doi:Doi 10.1126/Science.160.3829.770 (1968).

15 Vigne, P., Frelin, C., Cragoe, E. J. & Lazdunski, M. Ethylisopropyl-Amiloride - a New and highly potent derivative of amiloride for the inhibition of the Na^+/H^+ exchange system in various cell-types. *Biochem Bioph Res Com* **116**, 86-90, doi:Doi 10.1016/0006-291x(83)90384-4 (1983).

16 Charnock, J. S. & Post, R. L. Evidence of mechanism of ouabain inhibition of cation activated adenosine triphosphatase. *Nature* **199**, 910-911, doi:Doi 10.1038/199910a0 (1963).
